# Stable Zn Metal Anodes with Limited Zn-Doping in MgF_2_ Interphase for Fast and Uniformly Ionic Flux

**DOI:** 10.1007/s40820-021-00788-z

**Published:** 2022-01-22

**Authors:** Ji Young Kim, Guicheng Liu, Ryanda Enggar Anugrah Ardhi, Jihun Park, Hansung Kim, Joong Kee Lee

**Affiliations:** 1 Energy Storage Research Center, Korea Institute of Science and Technology, Hwarang-ro 14-gil 5, Seongbuk-gu, Seoul, 02792 Republic of Korea; 2 Department of Chemical and Biomolecular Engineering, Yonsei University, 50 Yonsei-ro, Seodaemun-gu, Seoul, 03722 Republic of Korea; 3 Department of Physics, Dongguk University, Seoul, 04620 Republic of Korea; 4 APC Technology, 108 68 Gangbyeonyeok-ro-4-gil, Gwangjin-gu, Seoul, 05116 Republic of Korea; 5 Department of Energy and Environmental Engineering, KIST School, Korea University of Science and Technology, Hwarang-ro 14-gil 5, Seongbuk-gu, Seoul, 02792 Republic of Korea

**Keywords:** Zinc metal battery, MgF_2_ layer, Limited zinc doping, Ion-transfer kinetic, Deposition guidance

## Abstract

The practical applications of aqueous Zn metal batteries are currently restricted by the inherent drawbacks of Zn such as the hydrogen evolution reaction, sluggish kinetics, and dendrite formation. To address these problems, herein, a limitedly Zn-doped MgF_2_ interphase comprising an upper region of pure, porous MgF_2_ and a lower region of gradient Zn-doped MgF_2_ is achieved via radio frequency sputtering technique. The porous MgF_2_ region is a polar insulator whose high corrosion resistance facilitates the de-solvation of the solvated Zn ions and suppression of hydrogen evolution, resulting in Zn metal electrodes with a low interfacial resistance. The Zn-doped MgF_2_ region facilitates fast transfer kinetics and homogeneous deposition of Zn ions owing to the interfacial polarization between the Zn dopant and MgF_2_ matrix, and the high concentration of the Zn dopant on the surface of the metal substrate as fine nuclei. Consequently, a symmetric cell incorporating the proposed Zn metal exhibits low overpotentials of ~ 27.2 and ~ 99.7 mV without Zn dendrites over 250 to 8000 cycles at current densities of 1.0 and 10.0 mA cm^−2^, respectively. The developed Zn/MnO_2_ full cell exhibits superior capacity retentions of 97.5% and 84.0% with average Coulombic efficiencies of 99.96% after 1000 and 3000 cycles, respectively.

**Supplementary Information:**

The online version contains supplementary material available at 10.1007/s40820-021-00788-z.

## Highlights

- A limitedly Zn-doped MgF_2_ passivation layer consisting of a porous pure MgF_2_- and gradient Zn-doped MgF_2_-regions was developed for stable Zn metal anodes with excellent reversibility to 8000 cycles at 10.0 mA cm^−2^.
- The MgF_2_ region facilitates facile de-solvation of Zn ions and effective hydrogen evolution reaction suppression.
- The Zn-doped MgF_2_ region enhances transfer kinetics and homogeneous deposition of Zn ions by interfacial polarization between Zn dopant and MgF_2_ matrix, and high concentration of the Zn dopant as fine nuclei.

**Supplementary Information:**

The online version contains supplementary material available at 10.1007/s40820-021-00788-z.

## Graphical abstract

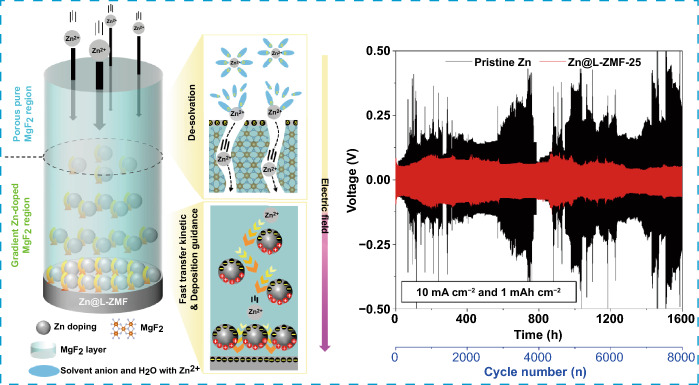

## Introduction

Zinc-based rechargeable batteries have recently attracted significant attention owing to their high safety achieved by utilizing aqueous electrolyte systems that reduce flammability [1–4]. Moreover, Zn metal anodes have attractive electrochemical properties such as a high theoretical capacity (820 mAh g^−1^, 5855 mAh cm^−3^) [5], low redox potential (− 0.76 V vs. SHE) [6], and low electrical resistivity (59 nΩ m) [7]. However, they have some disadvantages as electrodes owing to intrinsic problems such as the hydrogen evolution reaction (HER), susceptibility to corrosion, and Zn dendrite formation [8–10]. These critical limitations inhibit the stable reversibility of Zn metal anodes, resulting in a deterioration in the electrochemical performance of Zn metal batteries [11]. The corrosion and HER reactions are sustained and accelerated by the continuous growth of Zn dendrites, which increase the available surface area for the occurrence of side reactions and promote excessive solid electrolyte interphase (SEI) formation [12]. Therefore, the effective suppression of the Zn dendrite formation is a critical issue for stabilizing Zn metal anodes. In addition, divalent Zn^2+^ ions tightly bind to neutral ions or anions from the electrolyte owing to their high electric charge density [13, 14]. Solvated Zn ion encounters some difficulties passing through the interface between the Zn electrode and the electrolyte, leading to slow diffusion of Zn ions and a high interfacial resistance.

Various strategies have been employed to prevent the formation of Zn dendrites on Zn metal anodes and improve the ion transfer kinetics, such as modifying the electrolyte composition [13, 15], employing a 3D current collector [16, 17], and coating a passivation layer on the Zn metal surface [18–22]. The utilization of passivation layers for Zn metal anodes is considered a breakthrough because other strategies that modify the electrolyte and current collector are still limited in achieving complete isolation between the electrolyte and the electrode. Furthermore, the spontaneous formation of an SEI by the continuous exposure of the Zn metal to the electrolyte inhibits Zn ion diffusion. Recently, many beneficial surface coatings for Zn metal anodes, such as ZnO [18], ZrO_2_ [19], ZnS [20], ZnF_2_ [21], and kaolin (Al_2_Si_2_O_5_(OH_4_)) layers [22], prepared by in situ synthesis or slurry casting processes, not only function as physical barriers but also improve the Zn ion diffusion kinetics owing to the space charge polarization inside the coating layer. However, the micron-scale thickness of the abovementioned coating layers causes a high interfacial resistance and nucleation overpotential due to their low electron- and ion-conductivities, which can interrupt the transfer of Zn ions and cause a morphological change in the coatings. To maximize the transfer kinetics of the Zn ions in the coating layer, the thickness of the coating layers should be delicately adjusted at the nanoscale level.

**Scheme 1:**
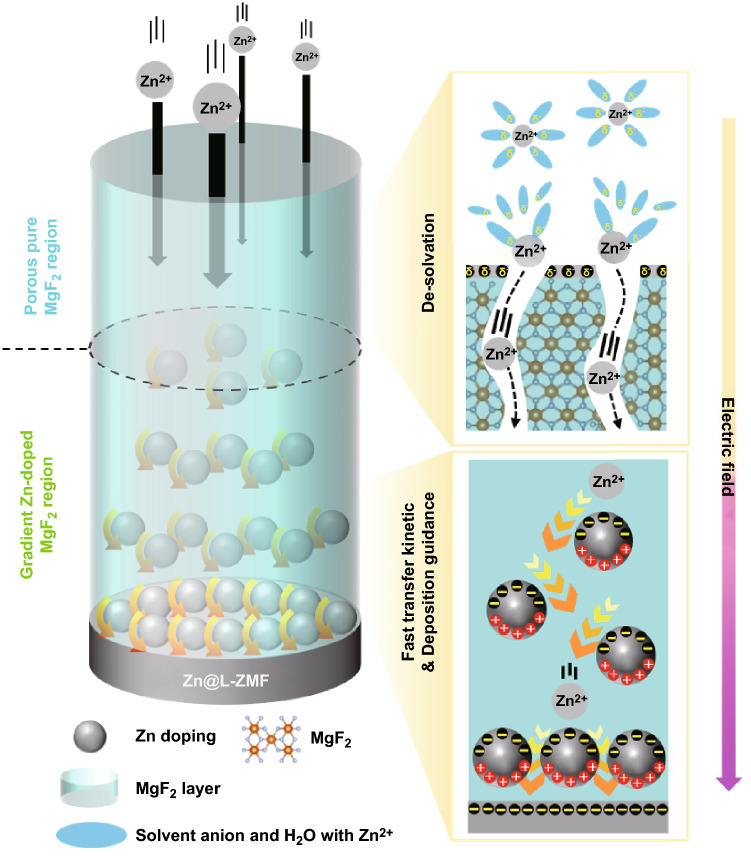
Illustration of the limitedly Zn-doped MgF_2_ thin passivation layer on Zn metal, with a schematic depiction of Zn ion migration through both the porous pure MgF_2_ region and gradient Zn-doped MgF_2_ region during the Zn ion plating process

Herein, a limitedly Zn-doped MgF_2_ (L-ZMF) passivation thin layer was synthesized on the surface of a Zn metal anode via a radio frequency sputtering technique. The passivation layer comprised an upper region of porous pure MgF_2_ and a lower region of gradient Zn-doped MgF_2_ (Scheme 1). The thermodynamically polar and electrochemically stable pure MgF_2_ region with evenly dispersed pores as pathways for the Zn ions can relieve the de-solvation barrier of the Zn ions and improve the HER resistance of the Zn metal, thereby lowering the interfacial resistance of the Zn metal electrode. Additionally, the distinctively gradient Zn-doping conformation formed by the interdiffusion mechanism during the sputtering process [23–27]; enhances the Zn ion transfer kinetics owing to an electrostatic driving force caused by Maxwell–Wagner polarization between Zn-doping and MgF_2_ matrix [19, 28]; it also guides the Zn ion deposition by the high concentration of doping of fine Zn nucleation centers on the surface of the Zn metal substrate [29, 30]. Consequently, the L-ZMF passivation layer coated Zn metal (Zn@L-ZMF) is an ideally stable Zn metal anode for Zn-based high-performance batteries.

## Experimental Methods

### Preparation of the Limitedly Zn-Doped MgF_2_ Passivation Layer on Zn Metal

Commercially available Zn metal was compressed to a thickness of 100 μm, washed with ethanol (50 mL) and D.I. water (50 mL) to remove organic impurities and dust, and transferred to the chamber of a radio frequency sputtering system equipped with an MgF_2_ target. The base and working pressures were maintained at 2.5 × 10^−6^ and 2.0 × 10^−2^ Torr, respectively. Ar gas at a flow rate of 25 sccm was used as the RF plasma source. The RF generator provided 100 W of power with a reflection power of less than 2 W. The thickness of the MgF_2_ passivation layer was controlled by adjusting the sputtering time to 5, 10, and 20 min.

### Material Characterization

The thickness and surface morphologies of Zn@L-ZMF electrodes were investigated using field-emission scanning electron microscope (FE-SEM, Inspect F, FEI). The chemical composition of the surface and bulk of the Zn metal were demonstrated by X-ray photoelectron spectroscopy (XPS, PHI 5000 VersaProbe, Ulvac-PHI). Cross-sectional samples were obtained using a focused ion beam milling system (Nova 600, FEI) with a Ga ion source. Field-emission-transmission electron microscope (FE-TEM, Talos F200X, FEI) coupled with energy-dispersive X-ray spectroscopy (EDS) was used to obtain the high-resolution TEM (HR-TEM) images, fast Fourier transform (FFT) patterns, and elemental maps. The contact angle between the Zn metal and aqueous electrolyte was measured using a contact angle goniometer system (L2004A1, Ossila). Crystallographic information was obtained using X-ray diffraction (XRD, Rigaku, Ultima IV) with a monochromatic Cu Kα source at a scan rate of 2° min^−1^.

### Electrochemical Characterization

Symmetric CR2032 cells with a glass fiber filter and a 1.0 mol L^−1^ zinc trifluoromethanesulfonate (Zn(OTf)_2_, 100 mL) or zinc sulfate monohydrate (ZnSO_4_·H_2_O, 100 mL) aqueous electrolyte solution were fabricated using Zn electrodes with a diameter of 14 mm as both the working and counter electrodes. The voltage profiles of the symmetric cell were measured at current densities of 0.5, 1.0, 3.0, 5.0, and 10.0 mA cm^−2^ with a constant areal capacity of 1.0 mAh cm^−2^. The Tafel polarization (TP, VSP-300 potentiostat, Bio-Logic Science Instruments) of the Zn metal was observed at a scan rate of 3.0 mV s^−1^ using a three-electrode system with a working electrode consisting of the Zn metal sample, with a graphite rod, saturated calomel electrode (SCE), and 1.0 mol L^−1^ Zn(OTf)_2_ aqueous solution (500 mL) as the working electrode, counter electrode, reference electrode, and electrolyte, respectively. Electrochemical impedance spectroscopy (EIS, VSP-300 potentiostat, Bio-Logic Science Instruments) of the symmetric cell was performed at an amplitude of 5 mV in the frequency range of 1 to 10 MHz. Chronoamperograms of the symmetric cell were measured at an overpotential of 25 mV for 4000 s. The Coulombic efficiency (CE) of Zn plating/stripping was estimated using a Ti/Zn half-cell which was operated at a current density of 1.0 mA cm^−2^ for 1 h during Zn plating on the Ti foil and reversibly at the same current density until 0.5 V (vs. Zn/Zn^2+^) during Zn stripping of the plated Zn on the Ti foil.

The Zn/MnO_2_ battery was fabricated with an α-MnO_2_ cathode and a 1.0 M Zn(OTf)_2_ + 0.1 M manganese sulfate (MnSO_4_) aqueous electrolyte solution (100 mL). MnSO_4_ was added to suppress the dissolution of Mn^2+^ from the MnO_2_ cathode. Cyclic voltammetry (CV) and the galvanostatic intermittent titration technique (GITT) were performed on the Zn/MnO_2_ battery using an electrochemical workstation (VSP-300 potentiostat, Bio-Logic Science Instruments). The rate and long-term cycling tests were performed on the Zn/MnO_2_ battery using a Maccor battery tester (MACCOR series-4000) in the voltage range of 1.0 to 1.8 V (vs. Zn/Zn^2+^).

## Results and Discussion

### Morphology and Composition of the L-ZMF Passivation Layer

**Fig. 1:**
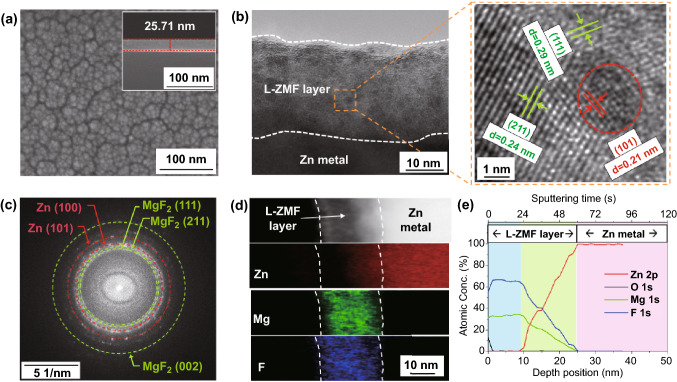
**a** Top-view and cross-sectional SEM images of Zn@L-ZMF-25. **b** Cross-sectional TEM-image and **c** FFT pattern of the L-ZMF layer of Zn@L-ZMF-25. **d** Elemental TEM-maps and **e** depth XPS-profile for Zn@L-ZMF-25.

Even though the side reactions of Zn corrosion and HER are effectively suppressed as MgF_2_ is an electrical insulator [31, 32], an excessively thick passivation layer inhibits the diffusion of the Zn ions to the Zn metal anode. Therefore, the L-ZMF thickness on the Zn metal surface was optimized by varying the radio frequency sputtering time. Surface SEM images of the Zn metal coated by L-ZMF layer for 10 min show a layer thickness of approximately 25 nm and a porous surface morphology (Fig. 1a). The SEM images of passivation layer with sputtering times of 5 and 20 min show a total thickness of about 13 and 40 nm, respectively (Fig. S1). Thus, the samples synthesized with sputtering times of 5, 10, and 20 min, were denoted as “L-ZMF-13,” “L-ZMF-25,” and “L-ZMF-40,” respectively.

The L-ZMF interphase, in which a high Zn dopant concentration was accumulated adjacent to the Zn metal substrate owing to the interdiffusion mechanism, was observed by HR-TEM and XPS. During the sputtering process, a few vacant spaces in the deposited thin film can be formed under the plasma environment. The Zn metal substrate is attacked by collision with plasma-activated Ar and sputtered MgF_2_. The activated Zn atoms diffuse by an interdiffusion phenomenon and settle as heteroatoms in the MgF_2_ matrix. The factors that affect interdiffusion are the atomic mobility, microstructure property, internal stress, and deposition conditions. The first three factors depend on the materials of the sputtered target and substrate. However, the deposition conditions can be adjusted by the researcher. These include the working pressure, temperature, and plasma power. Based on our experimental scope, the interdiffusion length of Zn-doping into MgF_2_ matrix is limited to 15 nm from the Zn metal substrate (Fig. S2) [33, 34]. In addition, the HR-TEM image and FFT pattern of the L-ZMF-25 layer demonstrate the co-existence of the MgF_2_ matrix and ~ 2.5 nm Zn metal dopant phase (Fig. 1b, c). The Zn metal dopant with a d-spacing of 0.21 nm indexed to the (101) plane was surrounded by MgF_2_ matrix with d-spacings of 0.24 and 0.29 nm indexed to the (211) and (111) planes, respectively [35–37].

Elemental maps of the Zn dopant distribution in L-ZMF-25 (Fig. 1d) show that the Zn content gradually increased from the surface to the Zn substrate, while the Mg and F contents gradually decreased. The high concentration of the nano-sized Zn dopant on the surface of the Zn metal substrate acted as fine nuclei for the homogeneous deposition of Zn ions. Quantification of this gradation phenomenon was also achieved by XPS analysis (Fig. 1e). First, only Mg and F were found at a depth of 10 nm with a constant atomic ratio of 1:2, indicating the presence of MgF_2_ [38]. Below the pure MgF_2_ region, the atomic concentrations of both Mg and F began to decrease gradually from a depth of 15 nm while the concentration of Zn increased, indicating the maximum diffusion length of the Zn nanoparticles into the MgF_2_ matrix from the Zn metal substrate during the RF sputtering process.

The XPS spectra of Zn 2p, Mg 1 s, and F 1 s were measured at various depths in the Zn@L-ZMF-25 electrode. The intensities of the peaks in the Zn 2p spectrum indicate that the quantity of metallic Zn gradually increased in the X-ray sputtering depth range of 10–25 nm, which is within the Zn-doped MgF_2_ region (Fig. S3a). In the Mg 1 s and F 1 s spectra, the peak intensities corresponding to MgF_2_ gradually decreased (Figs. S3b, c). Considering the mechanism by which the L-ZMF layer is formed, after reaching the limiting thickness of the Zn-doped MgF_2_ region, pure MgF_2_ was additionally deposited with further sputtering time. Likewise, both the L-ZMF-13 layer with a thickness of 13 nm having only a gradient Zn-doped MgF_2_ region and the L-ZMF-40 layer with a 25 nm-thick MgF_2_ region and a 15 nm-thick gradient Zn-doped MgF_2_ region were demonstrated (Fig. S4). Therefore, the L-ZMF layer provides a unique structural base exhibiting the electrically insulating polarity of pure MgF_2_, the Maxwell–Wagner polarization of Zn-doped MgF_2_ matrix, a high concentration of fine Zn-doping nuclei to realize anti-corrosion, fast ion transfer kinetics, and uniform ion-deposition guidance for Zn metal electrodes.

### Effect of the L-ZMF Thickness on Corrosion Resistance, Transference Number, and Nucleation Overpotential

**Fig. 2:**
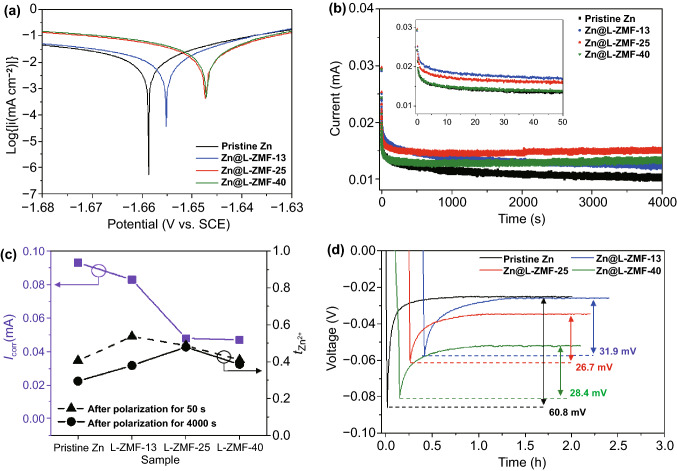
**a** Tafel polarization curves and **b** chronoamperograms of symmetric cells at a constant overpotential of 25 mV. **c** Summary for the corrosion current and transference number at the 50th and 4000th s. **d** Nucleation overpotential at a current density of 0.2 mA cm^−2^ with an areal capacity of 0.4 mAh cm^−2^ for pristine Zn, Zn@L-ZMF-13, Zn@L-ZMF-25, and Zn@L-ZMF-40

To verify the above inference and optimize the thickness of the L-ZMF passivation layers, the preservative effect, Zn ion transference number (${t}_{{Zn}^{2+}}$), and nucleation overpotential of the Zn@L-ZMF electrodes were examined. First, as shown in Fig. 2a and Table S1, the corrosion current of the Tafel polarization was inversely proportional to the sputtering time, and the corrosion potential increased with the thickness of the L-ZMF layers from − 1.660 to − 1.647 V (vs. SCE). After coating with L-ZMF, the corrosion resistance of the Zn@L-ZMF electrode against the side reactions, e.g., the HER, was enhanced and reached its limit at a sputtering time of 10 min.

The transference number was estimated to identify the Zn ion transferability of the L-ZMF passivation layer [20, 21, 39]. The Zn transference number (${t}_{{Zn}^{2+}}$) can be calculated by measuring the initial and steady state current and resistance of a symmetric cell under constant polarization. The Bruce–Vincent method defines the transference number as the current ratio between the initial and steady state because the interfacial composition and resistance constantly change at the interface of the electrode in a real battery system [40, 41]; that is, a high transference number corresponds to fewer compositional and low resistance changes in the electrode interphase, resulting in fast charge transfer kinetics.

During a polarization of 4000 s, the currents of the symmetric cells with pristine Zn and Zn@L-ZMF-13 electrodes gradually decreased, whereas those of the symmetric cells with Zn@L-ZMF-25 and Zn@L-ZMF-40 electrodes were essentially constant after 200 s (Fig. 2b). However, at the early polarization stage of 50 s, the highest current was observed in the symmetric cell with Zn@L-ZMF-13 electrodes. To explain this phenomenon, as analyzed by the charge transfer resistance (*R*_ct_, Table S2) calculated from EIS (Fig. S5), the interfacial resistances of all symmetric cells were barely altered after polarization for 50 s, enabling the conductivity of the Zn ions inside the respective L-ZMF layers to be estimated while maintaining the intact condition of the layers. During the first 50 s, the highest ${t}_{{Zn}^{2+}}$ of 0.537 (Fig. 2c) was observed in the Zn@L-ZMF-13 electrode, indicating that the Zn-doped MgF_2_ region enhanced the Zn transfer kinetics owing to the interfacial Maxwell–Wagner polarization between the Zn dopant and MgF_2_ matrix under a given electric field [21].

As the polarization time increased, the interfacial resistances of the symmetric cells with pristine Zn and Zn@L-ZMF-13 electrodes increased, thereby decreasing ${t}_{{Zn}^{2+}}$; this may be attributed to the morphological and compositional changes of the Zn electrodes arising from spontaneous side reactions with an aqueous electrolyte [42]. In contrast, the interfacial resistances of symmetric cells with Zn@L-ZMF-25 and Zn@L-ZMF-40 electrodes decreased during the first 50 s. After 4000 s, the highest ${t}_{{Zn}^{2+}}$ of the four samples (0.454) was eventually drawn in the Zn@L-ZMF-25 electrode. The ${t}_{{Zn}^{2+}}$ of the Zn@L-ZMF-40 electrode was lower than that of the Zn@L-ZMF-25 electrode because it has a thicker MgF_2_ layer. Consequently, even though the Zn-doped MgF_2_ region considerably enhanced the Zn transfer kinetics, it is necessary to optimize the thickness of the MgF_2_ region to prevent undesirable side reactions in the intact L-ZMF passivation layer.

To further investigate the relationship between the morphological change and the transference number, the surface morphology of each Zn metal electrode was confirmed after galvanostatic cycling. The symmetric cell with pristine Zn electrodes exhibited a fluctuating and unstable voltage profile with high overpotential during the repetitive Zn plating and stripping (Fig. S6a). In contrast, the symmetric cells with Zn@L-ZMF electrodes displayed stable voltage profiles with average overpotentials of 15.3, 20.1, and 63.2 mV for the Zn@L-ZMF-13, Zn@L-ZMF-25, and Zn@L-ZMF-40 electrodes, respectively. Correspondingly, a very rough surface was formed on the pristine Zn metal owing to the formation of Zn dendrites (Fig. S6b). The Zn@L-ZMF-13 electrode showed a mildly uneven surface due to spontaneous side reactions with the aqueous electrolyte, which was confirmed by the Tafel plot (Fig. 2a). Additionally, the natural and artificial layers were too weak and too thin, respectively, and thus easily broken by the growth of Zn dendrites and occurrence of side reactions, leading to cracks and the formation of a new SEI layer. During long-term polarization, the newly and continuously formed thickened SEI layer interrupted the diffusion of the solvated Zn ions into the Zn metal electrode, leading to a low Zn ion transference number and correspondingly sluggish kinetics. In contrast, no Zn dendrites were found in either Zn@L-ZMF-25 or Zn@L-ZMF-40 owing to the porous and Zn-doped MgF_2_ regions in their complete L-ZMF passivation layers, which prevent side reactions and improve the Zn ion transfer kinetics.

In asymmetric cells, the L-ZMF passivation layers were found to significantly alleviate the nucleation overpotential toward Zn ions compared to that of pristine Zn metal with the nucleation overpotential of 60.8 mV (Fig. 2d). Even though the Zn@L-ZMF-13 exhibited the lowest onset potential, its interfacial resistance continuously increased owing to spontaneous side reactions between the exposed Zn metal in the Zn-doped MgF_2_ layer and the aqueous electrolyte, resulting in a higher nucleation overpotential than the Zn@L-ZMF-25 and Zn@L-ZMF-40 electrodes (26.7 and 28.4 mV, respectively). The thickness of the L-ZMF passivation layer is determined by a trade-off among the electrochemical parameters such as the Zn corrosion tendency, Zn transference number, and nucleation overpotential. As a result, the Zn@L-ZMF-25 electrode, with a L-ZMF-passivation-layer-coated Zn metal, was found to be optimal.

### Investigation of Interfacial Properties and Symmetric Cell Performance of the Pristine Zn and Zn@L-ZMF-25 Electrodes

**Fig. 3:**
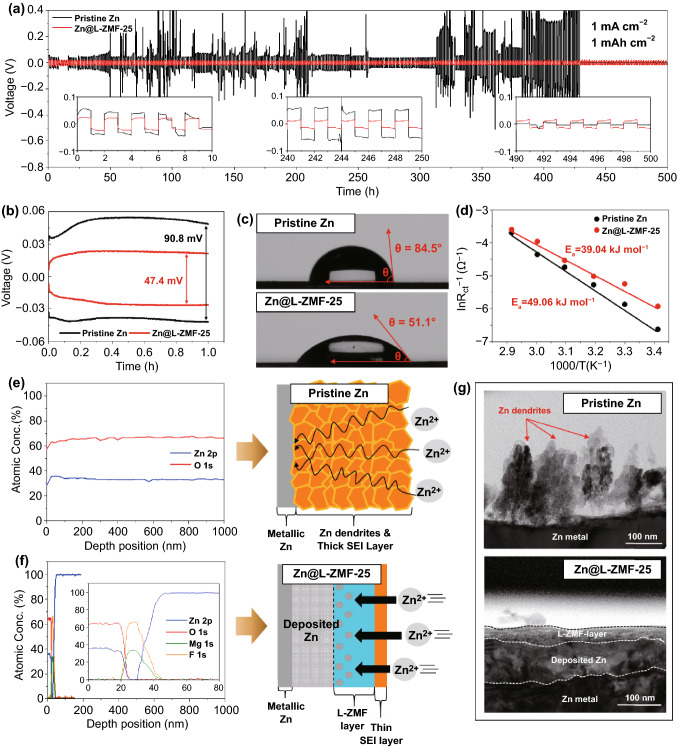
Voltage profiles of **a** long-term and **b** single Zn plating and stripping performance of the symmetric cell at a current density of 1.0 mA cm^−2^ with an areal capacity of 1.0 mAh cm^−2^. **c** Contact angle images and **d** linear plots based on the Arrhenius equation and the, respectively, derived activation energies before the cycling test. Post-mortem SEI-thicknesses calculated by XPS depth profiles for **e** pristine Zn and **f** Zn@L-ZMF-25 electrodes. **g** Comparison of the cross-sectional images between the pristine Zn and Zn@L-ZMF-25 electrodes after Zn plating

A symmetric cell with Zn@L-ZMF-25 electrodes shows a more stable voltage profile with a lower overpotential than a cell with pristine Zn electrodes over 250 cycles owing to its superior anti-corrosion properties, enhanced ion transfer kinetics, and lack of dendrites (Fig. 3a). The volume of the symmetric cell with pristine Zn was considerably expanded after 250 galvanostatic cycles compared with that in the symmetric cell before cycling (Fig. S7), which was attributed to the HER on the pristine Zn metal. The volume expansion of the symmetric cell with Zn@L-ZMF-25 electrodes was insignificant, indicating that the HER on the Zn@L-ZMF-25 electrode was also significantly inhibited. Furthermore, a separator of glass fiber filters was employed to observe the changes in the Zn electrode properties after cycling. Rich dark debris was found in the symmetric cell with pristine Zn metal electrodes (Fig. S8a); the particles were proven to be “dead Zn” and by-products such as ZnO, Zn(OH)_2_, and ZnSO_4_ (Fig. S8b), resulting from the growth of Zn dendrites and side reactions with the electrolyte. On the contrary, the separator used in the symmetric cell with Zn@L-ZMF-25 electrodes presented a clean surface with few by-products, indicating that the Zn dendrite growth and side reactions were effectively suppressed.

The expanded voltage profiles of the symmetric cell with pristine Zn metal electrodes eventually attained an internal short-circuit, which featured a voltage of nearly 0 V (Fig. 3a). Conversely, a stable voltage profile was observed for 250 cycles in the symmetric cell with Zn@L-ZMF-25 electrodes with a low overpotential hysteresis of 47.4 mV (Fig. 3b). To understand the mechanism of the performance enhancement, the interfacial characteristics and morphological changes of the optimized L-ZMF passivation layer in aqueous electrolyte were electrochemically and thermodynamically studied and compared with those of the pristine Zn electrode. The Nyquist plot of Zn@L-ZMF-25 showed a smaller semi-circle than that of pristine Zn at the initial state at 20 °C (Fig. S9). The overpotential is affected by the overall resistances including the interface resistance (${R}_{sf}$) and charge transfer resistance (${R}_{ct}$). Because the contact angle of Zn@L-ZMF-25 with an aqueous electrolyte was significantly lower (Fig. 3c), the improved permeability of the aqueous electrolyte indicates a low interfacial resistance in Zn@L-ZMF-25.

Notably, the charge transfer resistance mainly comes from the Zn ion transfer process until the Zn ions are reduced to metallic Zn. Two perspectives could be taken into account to explain the charge transfer resistance, which are related to the transfer of the electrons and Zn ions [18, 43]. The electron transfer at the interface between the Zn metal and current collector could be neglected when using the same Zn metal and current collector (stainless steel) with high electric conductivities of 1.67 × 10^7^ and 1.45 × 10^6^ S m^−1^, respectively. The Zn ion transfer could be considered both at the interphase and at the surface of the Zn electrode. The Zn ion transfer kinetics at the L-ZMF interphase region (as already identified by the transference number in Fig. 2) was studied in addition to the Zn ion transferability at the entrance of the Zn electrodes by the activation energy ($E_{a}$), which is an important criterion to evaluate the de-solvation ability [43–45]. Moreover, the de-solvation of the cations is the rate-determining step in the charge transfer into the active material affecting the overall charge transfer resistance [46]. Thus, both the activation energy and charge transfer resistance comply with Arrhenius Eq. (1) at a given temperature as follows:

1 $$\ln R_{{{\text{ct}}}} = - \frac{{E_{a} }}{R}\left( \frac{1}{T} \right) + \ln A$$

where $R_{{{\text{ct}}}}$ is the charge transfer resistance,$E_{a}$ is the activation energy of the de-solvation process, *R* is the ideal gas constant, *T* is the temperature in Kelvin, and *A* is the frequency factor.

To estimate the activation energy, the charge transfer resistance of the Zn ions was measured via EIS analysis of the symmetric cell at various temperatures (Fig. S10 and Table S3). The measured charge transfer resistances were linearly plotted against the operation temperatures (Fig. 3d). Based on the Arrhenius relationship, the activation energy calculated by the slope of this plot for Zn@L-ZMF-25 was 39.04 kJ mol^−1^, which was lower than that of pristine Zn (49.06 kJ mol^−1^), suggesting that the highly polar L-ZMF passivation layer contributed to the facile de-solvation of the Zn ions [47–49], thereby lowering the charge transfer resistance. The improved ability for de-solvation of the L-ZMF layer might be attributed to a physical property such as polarization of MgF_2_. MgF_2_ can be polarized due to the difference of electronegativity between Mg and F [50]. This facilitates the facile transfer of Zn ions into the porous MgF_2_ layer because the solvated Zn ions can be liberated due to the electrical repulsion between polarized F and anions.

Comparing the chemical compositions at various depths after 250 galvanostatic cycles (Fig. 3e, f), Zn@L-ZMF-25 exhibited an extremely thin SEI layer, essentially maintaining the established L-ZMF layer. This indicates that the L-ZMF-25 passivation layer significantly inhibits the HER and maintains superior electrochemical performance. In contrast, the pristine Zn metal had a large SEI layer with a thickness of more than 1 μm, which consisted entirely of Zn(OH)_2_ (Fig. S11). The main by-product is dependent on the salts in the aqueous electrolyte; in ZnSO_4_-based electrolyte, the main product is Zn_4_SO_4_(OH)_6_ hydrate [51, 52], whereas in Zn(CF_3_SO_3_)_2_–based electrolyte, Zn(OH)_2_ is mainly formed [53, 54]. Importantly, the electrically insulated Zn(OH)_2_ material is a representative by-product of the HER in Zn metal electrodes with aqueous electrolytes, and reduces their electrochemical performance. Unlike the rough surface morphology caused by severe dendritic growth on the pristine Zn metal, the even surface in the Zn@L-ZMF-25 was maintained after 250 galvanostatic cycles (Fig. S12).

To directly observe the dendrite-suppression mechanism in comparison with that shown in Fig. 3g, Zn metal was plated at a current density of 1.0 mA cm^−2^ for 10 h and located under the electron insulating L-ZMF passivation layer, which experimentally proved that the Zn ions could pass through the MgF_2_ region, resulting in a dendrite-free Zn metal electrode. In order to clearly demonstrate the underlying deposition of Zn ions near the L-ZMF layer, an expanded cross-sectional image of Zn@L-ZMF-25 was taken after Zn plating (Fig. S13a). Even though the MgF_2_ layer was porous, the plated Zn ions were located below the MgF_2_ layer due to its electrically insulating property. Furthermore, the L-ZMF layer remained intact after stripping the plated Zn with an areal capacity of 10 mAh cm^−2^ (Fig. S13b). The dendrite-free plating behavior of Zn ions was well reflected in the improved CE. At the pristine Ti electrode without a MgF_2_-based coating layer, the CE was significantly low and unstable. However, the Ti foil electrode coated by MgF_2_-based interphase (Ti@L-TMF-25) showed a superior CE of 98.8% after 100 cycles (Fig. S14). This can be attributed to the suppression of Zn dendrite growth, inhibiting the formation of dead Zn which is the main reason for capacity losses [12].

The superior rate capability of the Zn@L-ZMF-25 electrode was demonstrated at various current densities ranging from 0.5 to 10.0 mA cm^−2^ with a constant areal capacity of 1.0 mAh cm^−2^ (Fig. S15). Compared with that of the pristine Zn electrode, more stable voltage behavior was observed for the Zn@L-ZMF-25 electrode, with lower overpotential. In particular, the pristine Zn electrode resulted in a partial and complete short-circuit with a sudden voltage reduction and approximately zero voltage at current densities of 3.0 and 5.0 mA cm^−2^, respectively. However, a steady potential profile without any voltage fluctuation was recorded in Zn@L-ZMF-25 across all current densities. The long-term stability of Zn plating and stripping conducted at the extremely high current density of 10.0 mA cm^−2^ shows that Zn@L-ZMF-25 demonstrated enhanced stability to reversible Zn plating and stripping over 8000 cycles: thus, the long-term stability of Zn@L-ZMF-25 is greater than those of the Zn metal electrodes reported in the current literature (Table S4). The Zn(CF_3_SO_3_)_2_-based electrolyte is beneficial owing its higher ionic conductivity and bulky CF_3_SO_3_ anions, which decrease the number of hydrated Zn ions [13, 55]. However, in order to prove the compatibility of the L-ZMF layer regardless of the type of aqueous electrolyte used, we performed further experiments to confirm the long-term stability of Zn plating and stripping in ZnSO_4_-based aqueous electrolytes. Indeed, in 1.0 M ZnSO_4_ aqueous electrolyte, the symmetric cell with Zn@L-ZMF-25 demonstrated stable voltage behavior and a low overpotential over 500 cycles (Fig. S16), suggesting that the L-ZMF passivation layer is suitable for use with stable Zn metal electrodes in various aqueous electrolytes.

### Electrochemical Performance Comparison between the Pristine Zn/MnO_2_- and Zn@L-ZMF-25/MnO_2_-full Cells

**Fig. 4:**
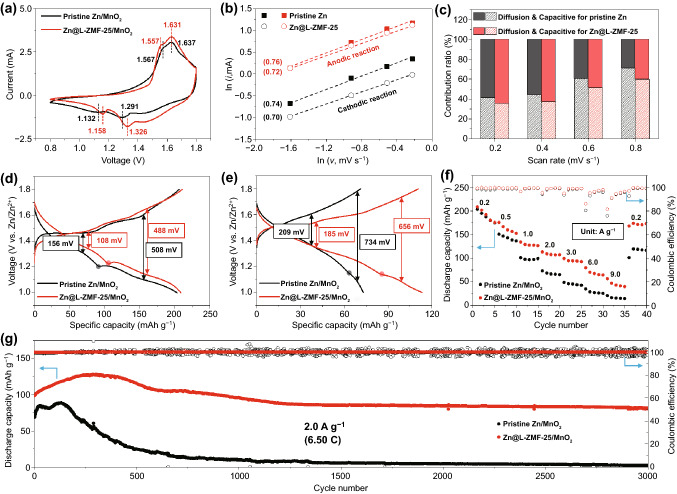
**a** Cyclic voltammetry (CV) curves of Zn/MnO_2_ full cells at a scan rate of 0.8 mV s^−1^. **b** Linear fitting of ln(*i*) as a function of ln(ν) and **c** contribution ratio of capacitance and diffusion-controlled behaviors for the Zn/MnO_2_ full cells with pristine Zn and Zn@L-ZMF-25 anodes. Discharge–charge voltage profiles at current densities of **d** 0.2 and **e** 2.0 A g^−1^. **f** Rate performance (unit: A g^−1^) and **g** capacity retention of the pristine Zn/MnO_2_ and Zn@L-ZMF-25/MnO_2_ full cells

To demonstrate the applicability of Zn@L-ZMF-25 as an anode material in practical energy storage systems, various electrochemical tests were conducted using a Zn/MnO_2_ battery. Before the cycling test, XRD of the synthesized MnO_2_ (Fig. S17) showed that the MnO_2_ nanowire possessed an alpha phase structure, obvious lattice fringes, and a FFT pattern related to α-MnO_2_. Compared to the pristine Zn/MnO_2_ cell, the Zn@L-ZMF-25/MnO_2_ cell exhibited a smaller voltage gap between the anodic and cathodic reactions in the CV curve owing to the low interfacial and charge transfer resistances of Zn@L-ZMF-25, as mentioned above (Fig. 4a). A clear redox peak and large area of the CV curves in the Zn@L-ZMF-25/MnO_2_ cell were exhibited at different scan rates (Fig. S18).

The Zn ion storage capability of MnO_2_ can be estimated using the power-law expression $i={av}^{b}$ (Fig. 4b), where *i* and *v* indicate the peak current and scan rate, respectively [56]. The charge storage mechanism incorporates both capacitive (*b* = 1) and diffusion (*b* = 0.5) controlled behaviors [57]. The *b* values of the two redox peaks of the Zn@L-ZMF-25/MnO_2_ cell were closer to 0.5 than that of the pristine Zn/MnO_2_ cell. The capacity contribution of the Zn/MnO_2_ batteries with pristine Zn and Zn@L-ZMF-25 anodes were visually distinguished by the capacitive and diffusion-controlled behaviors at all scan rates (Figs. S19 and S20). Accordingly, the contribution of the diffusion-controlled behavior to the capacity ratio of the Zn@L-ZMF-25/MnO_2_ cell was greater than that of the pristine Zn/MnO_2_ cell (Fig. 4c). Furthermore, the diffusion coefficient of the Zn@L-ZMF-25/MnO_2_ cell was higher than that of the pristine Zn/MnO_2_ cell, as calculated using the GITT test (Fig. S21). The overpotential and voltage plateau in the charge–discharge voltage profiles of the Zn@L-ZMF-25/MnO_2_ cell were lower and longer than those in the profiles of the pristine Zn/MnO_2_ cell (Fig. 4d, e), indicating that the Zn@L-ZMF-25 anode could maintain the structural stability of the MnO_2_ cathode. In this respect, the crystallographic information of the MnO_2_ cathode after cycling of the Zn/MnO_2_ cells will be investigated below. The rate capability of the Zn/MnO_2_ full cells was compared with those of the pristine Zn and Zn@L-ZMF-25 (Fig. 4f). At current densities of 0.2 to 9.0 A g^−1^, the discharge capacity and CE of the Zn@L-ZMF-25/MnO_2_ cell were higher than those of the pristine Zn/MnO_2_ cell. When the current density returned to the initial level of 0.2 A g^−1^, the recovery ratio of the discharge capacity in the Zn@L-ZMF-25/MnO_2_ cell was approximately 95.7%, whereas that of the pristine Zn/MnO_2_ cell was approximately 57.8%.

The long-term cycling performance of Zn/MnO_2_ full cells was studied at a constant current density of 2.0 A g^−1^ (Fig. 4g). Compared to the initial discharge capacity of 68.8 mAh g^−1^ and poor cycling performance with a steep reduction after the 150^th^ cycle observed in the pristine Zn/MnO_2_ cell, the Zn@L-ZMF-25/MnO_2_ cell exhibited a higher initial discharge capacity of 98.8 mAh g^−1^, which was attributed to the low interfacial resistance of the Zn@L-ZMF-25 anode and the outstanding capacity retentions of 97.5% and 84.0% with an average CE of 99.96% after the 1000 and 3000 cycles, respectively. These results are superior to the reported long-term cycling performance of existing Zn/MnO_2_ full cells in the literature (Table S5).

After long-term cycling, the condition of the MnO_2_ cathode was investigated to determine the working mechanism. TEM images and elemental mapping (Fig. S22) revealed that slight agglomerations were presented on the MnO_2_ nanowires of the Zn@L-ZMF-25/MnO_2_ cell, whereas severe agglomerations were observed on the MnO_2_ nanowires of the pristine Zn/MnO_2_ cell. The agglomerations on the MnO_2_ cathode were composed of Zn-based by-products supplied by the wandering Zn nanoparticles due to fracture of the Zn dendrites, which could inhibit the permeation of the Zn ions into the MnO_2_ crystal, resulting in low cathodic capacity. In addition, HR-TEM images and FFT patterns (Fig. S23) show that the crystal structure of MnO_2_ in the Zn@L-ZMF-25/MnO_2_ electrode was maintained with clear lattice fringes. In contrast, the collapse of the MnO_2_ crystal was observed in the pristine Zn/MnO_2_ cell. The overpotential originating from the anode affected the potential change of the cathode, causing a phase transition in the inactive materials [58, 59]. Therefore, this study experimentally demonstrated that the Zn@L-ZMF-25 anode could effectively inhibit the phase transition of the MnO_2_ cathode and the formation of by-products due to the low overpotential and the absence of dendritic growth on the Zn anode.

## Conclusions

A Zn metal electrode with limited Zn-doping in the MgF_2_ interphase was prepared by a radio frequency sputtering technique. Owing to the interdiffusion of Zn metal into the MgF_2_ layer during the RF sputtering process, the deposited L-ZMF layer consists of two regions: a porous pure MgF_2_ region and gradient Zn-doped MgF_2_ region. The L-ZMF layer exhibits a constant thickness of 15 nm. The polar and insulating MgF_2_ layer effectively enabled the solvated Zn ions to be easily stripped, resulting from a low activation energy (${E}_{a}$) for Zn ion transfer, and enhanced corrosion resistance owing to the inhibition of side reactions such as the HER. Moreover, the Zn ions deposited beneath the passivation layer led to a dendrite-free Zn metal electrode layer by blocking electron transfer. The Zn-doped MgF_2_ region resulted not only in fast Zn transfer kinetics due to the interfacial polarization between the Zn dopant and the MgF_2_ layer based on the Maxwell–Wagner effect but also caused the uniform deposition of Zn ions. The high concentration of Zn dopant as fine nuclei of approximately 2.5 nm at the interface between the Zn metal and L-ZMF effectively reduced the nucleation overpotential. As an optimal Zn electrode with an L-ZMF thickness of 25 nm, the Zn@L-ZMF-25-based symmetric cell exhibited a significantly reduced overpotential and stable voltage behavior without any fluctuation in the range of 0.5–10.0 mA cm^−2^, even after 8000 cycles at the extremely high current density of 10 mA cm^−2^ with an areal capacity of 1.0 mAh cm^−2^. The Zn@L-ZMF-25/MnO_2_ full cell exhibited an outstanding rate performance and a long-term capacity retention. Moreover, excellent capacity retentions of 97.5% and 84.0% (with an average CE of 99.96%) were maintained after 1000 and 3000 cycles, respectively, at a current density of 2.0 A g^−1^. The significantly improved electrochemical performance of the Zn@L-ZMF-25/MnO_2_ full cell was attributed to the low interfacial resistance and rapid ion diffusion kinetics of the Zn metal anode caused by the L-ZMF passivation layer, and the prevention of crystal collapse in the MnO_2_ cathode.

## Supplementary Information

Below is the link to the electronic supplementary material.

Supplementary file1 (PDF 1714 kb)
