# Correction to: Stable Zn Metal Anodes with Limited Zn-Doping in MgF_2_ Interphase for Fast and Uniformly Ionic Flux

**DOI:** 10.1007/s40820-022-00814-8

**Published:** 2022-03-04

**Authors:** Ji Young Kim, Guicheng Liu, Ryanda Enggar Anugrah Ardhi, Jihun Park, Hansung Kim, Joong Kee Lee

**Affiliations:** 1 Energy Storage Research Center, Korea Institute of Science and Technology, Hwarang-ro 14-gil 5, Seongbuk-gu, Seoul, 02792 Republic of Korea; 2 Department of Chemical and Biomolecular Engineering, Yonsei University, 50 Yonsei-ro, Seodaemun-gu, Seoul, 03722 Republic of Korea; 3 Department of Physics, Dongguk University, Seoul, 04620 Republic of Korea; 4 APC Technology, 108 68 Gangbyeonyeok-ro-4-gil, Gwangjin-gu, Seoul, 05116 Republic of Korea; 5 Department of Energy and Environmental Engineering, KIST School, Korea University of Science and Technology, Hwarang-ro 14-gil 5, Seongbuk-gu, Seoul, 02792 Republic of Korea

## Correction to: Nano-Micro Lett. (2022) 14:46 https://doi.org/10.1007/s40820-021-00788-z

In the original version of the article affiliation 1 has been missed out for an author and affiliation 1 superscript has been added in this correction.

The Original article has been corrected.

Joong Kee Lee^1,5^

