# Supplementary material for: Stable Zn Metal Anodes with Limited Zn-Doping in MgF2 Interphase for Fast and Uniformly Ionic Flux
Source: Nanomicro Lett. 2022 Jan 22;14:46. doi: 10.1007/s40820-021-00788-z (PMC8783935; doi:10.1007/s40820-021-00788-z)
Supplement: Supplementary file 1 — Supplementary file1 (PDF 1714 kb) [file 40820_2021_788_MOESM1_ESM.pdf]

Supporting Information for

Stable Zn Metal Anodes with Limited Zn-Doping in MgF_2 Interphase for Fast and Uniformly Ionic Flux

Ji Young Kim[1, 2], Guicheng Liu[1, 3, *], Ryanda Enggar Anugrah Ardhi[1], Jihun Park[4], Hansung Kim[2], Joong Kee Lee[1, 5, *]

[1]Energy Storage Research Center, Korea Institute of Science and Technology, Hwarang-ro 14-gil 5, Seongbuk-gu, Seoul 02792, Republic of Korea

[2]Department of Chemical and Biomolecular Engineering, Yonsei University, 50 Yonsei-ro, Seodaemun-gu, Seoul 03722, Republic of Korea

[3]Department of Physics, Dongguk University, Seoul 04620, Republic of Korea

[4]APC Technology, 108 68 Gangbyeonyeok-ro-4-gil, Gwangjin-gu, Seoul 05116, Republic of Korea

[5]Department of Energy and Environmental Engineering, KIST School, Korea University of Science and Technology, Hwarang-ro 14-gil 5, Seongbuk-gu, Seoul 02792, Republic of Korea

*Corresponding authors. E-mail: log67@163.com or liuguicheng@dongguk.edu (G. Liu); leejk@kist.re.kr (J. K. Lee)

Supplementary Figures and Tables

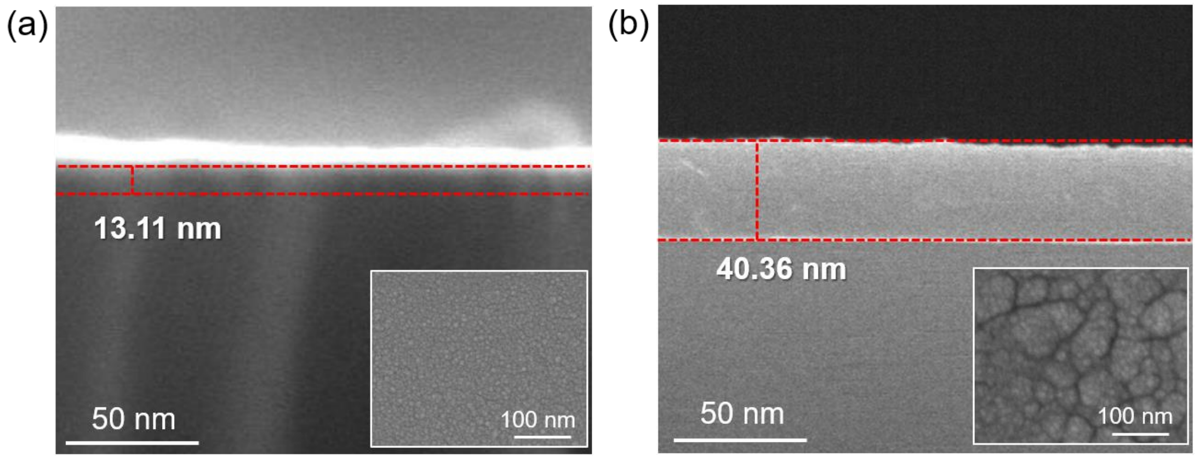

Fig. S1 Top-view and cross-sectional SEM images of MgF_2-coated Zn metal with sputtering times of (**a**) 5 min (Zn@L-ZMF-13) and (**b**) 20 min (Zn@L-ZMF-40)

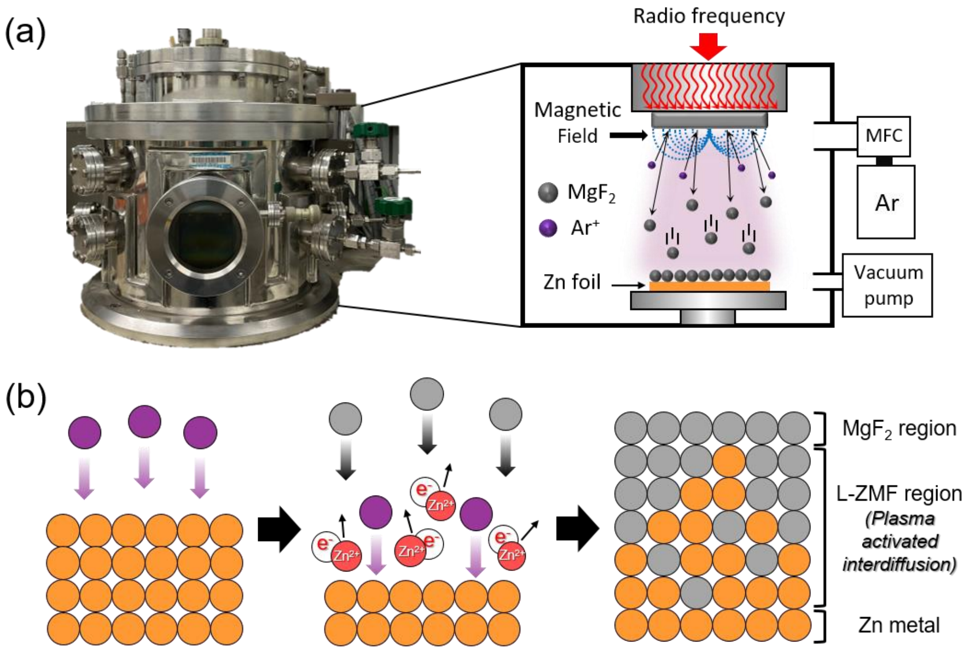

Fig. S2 Schematic illustration of (**a**) radio frequency sputtering system and (**b**) interdiffusion mechanism of Zn doping into deposited MgF_2 layer

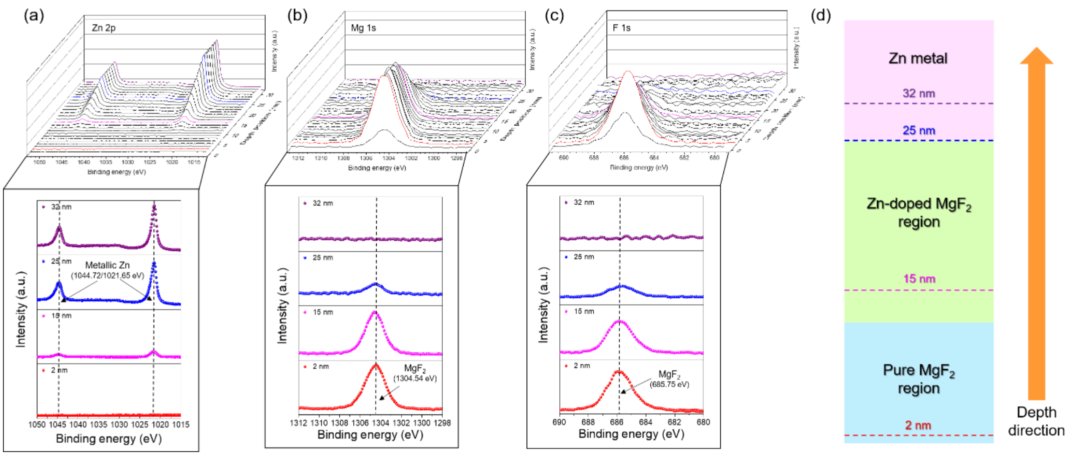

Fig. S3 X-ray photoelectron spectroscopy (XPS) spectra of (**a**) Zn 2p, (**b**) Mg 1s, and (**c**) F 1s of Zn@L-ZMF-25 at depths of 2, 15, 25, and 32 nm. (**d**) Schematic of the respective depths

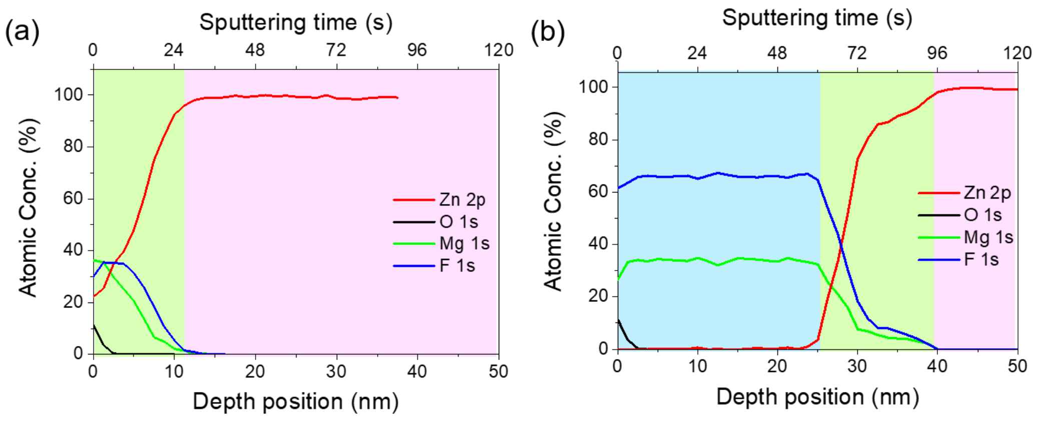

Fig. S4 Depth profiles of MgF_2-coated Zn metals with sputtering times of (**a**) 5 min (Zn@L-ZMF-13) and (**b**) 20 min (Zn@L-ZMF-40)

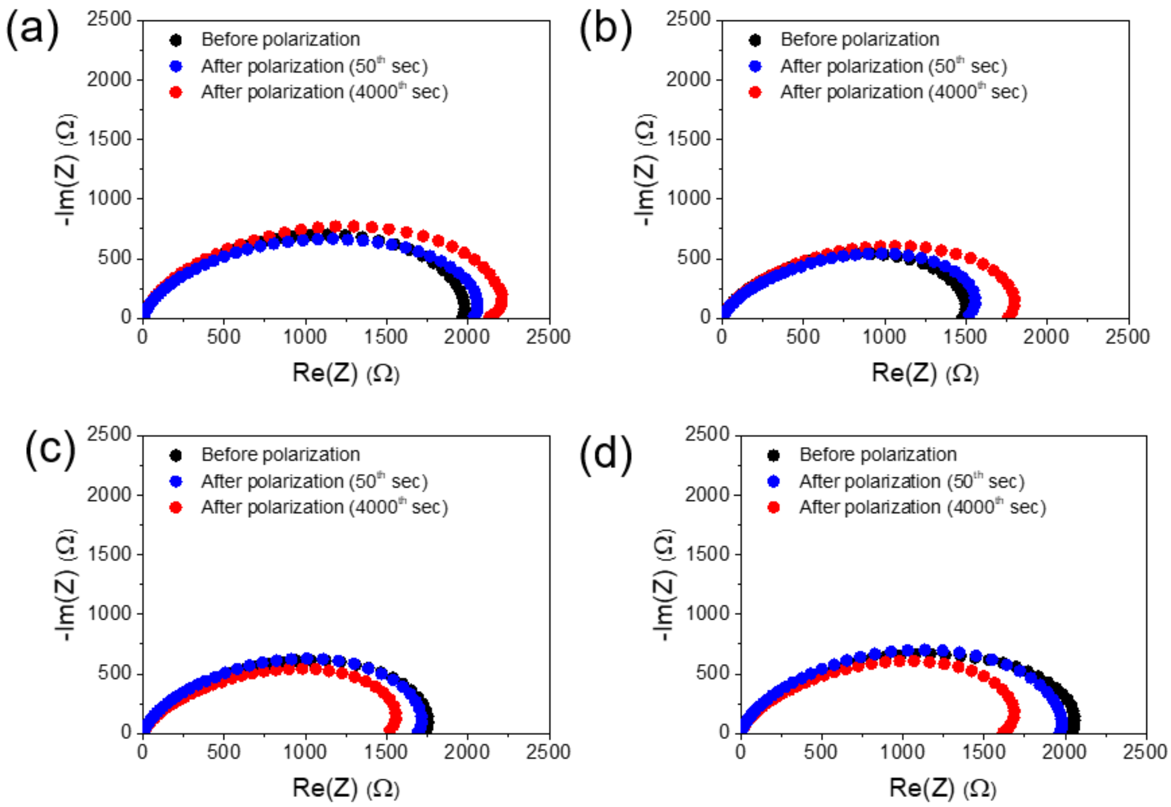

Fig. S5 Nyquist plots of symmetric cells with (**a**) pristine Zn, (**b**) Zn@L-ZMF-13, (**c**) Zn@L-ZMF-25, and (**d**) Zn@L-ZMF-40 electrodes before and after polarization

Note for Fig. S5: The Zn ion transference number ($t_{Zn^{2+}}$) was calculated via the Bruce–Vincent equation (Eq. S1).

$$t_{Zn^{2+}} = \frac{I_S\,(\Delta V - I_0 R_0)}{I_0\,(\Delta V - I_S R_S)} \qquad \text{(S1)}$$

where I_0 and I_S are the initial and steady-state currents, respectively. ΔV is the applied voltage of 25 mV. R_0 and R_S are the initial and steady-state resistances, respectively. The transference number is employed to compare the kinetics of Zn ion transfer at the interface between an aqueous electrolyte and a Zn metal electrode.

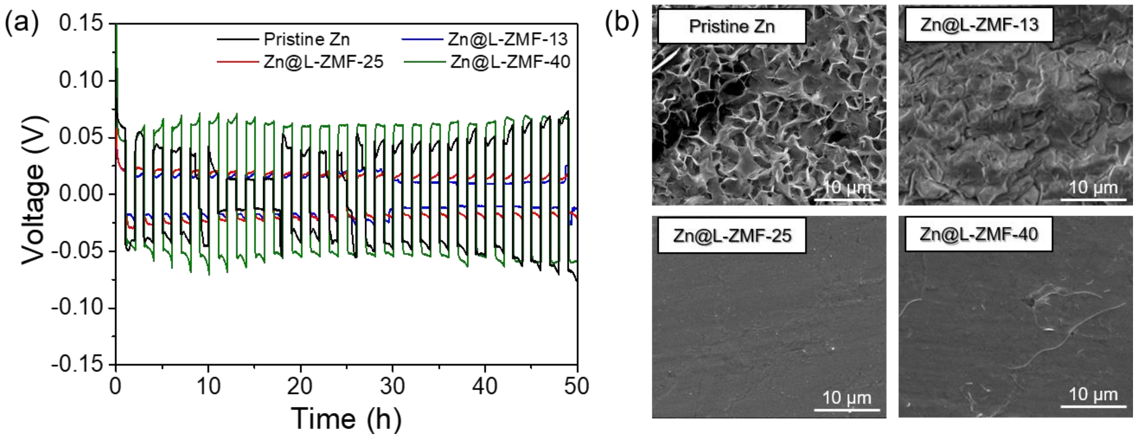

Fig. S6 (**a**) Galvanostatic cycling of symmetric cells at a current density of 1.0 mA cm^{-2} with an areal capacity of 1.0 mAh cm^{-2}. (**b**) Top-view SEM images of pristine Zn, Zn@L-ZMF-13, Zn@L-ZMF-25, and Zn@L-ZMF-40 electrodes after 25 galvanostatic cycles at a current density of 1.0 mA cm^{-2} with an areal capacity of 1.0 mAh cm^{-2}

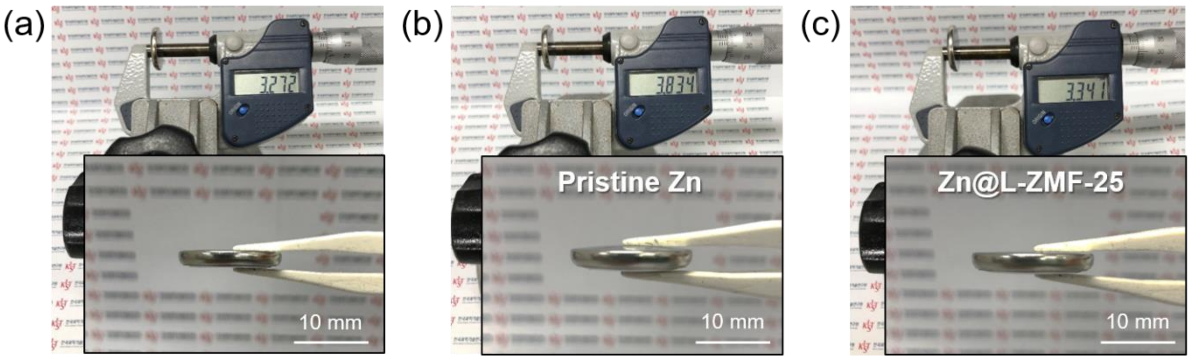

Fig. S7 Thicknesses of (**a**) newly assembled cell and symmetric cells with (**b**) pristine Zn and (**c**) Zn@L-ZMF-25 electrodes after 250 galvanostatic cycles at a current density of 1.0 mA cm^{-2} with an areal capacity of 1.0 mAh cm^{-2}

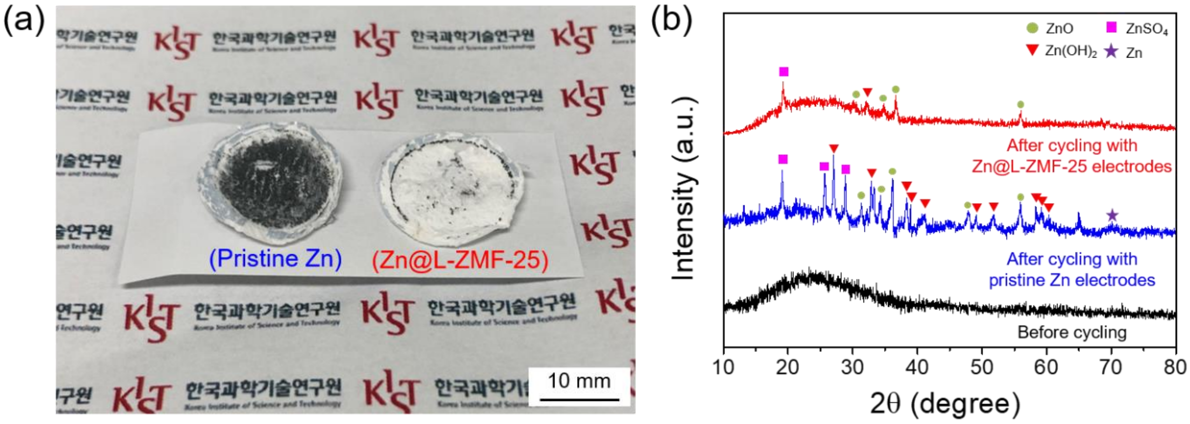

Fig. S8 (**a**) Photographs and (**b**) X-ray diffraction (XRD) patterns of the used glass fiber filter separators in the symmetric cells with (left) pristine Zn and (right) Zn@L-ZMF-25 electrodes after 250 galvanostatic cycles at 1.0 mA cm^{-2} with an areal capacity of 1.0 mAh cm^{-2}

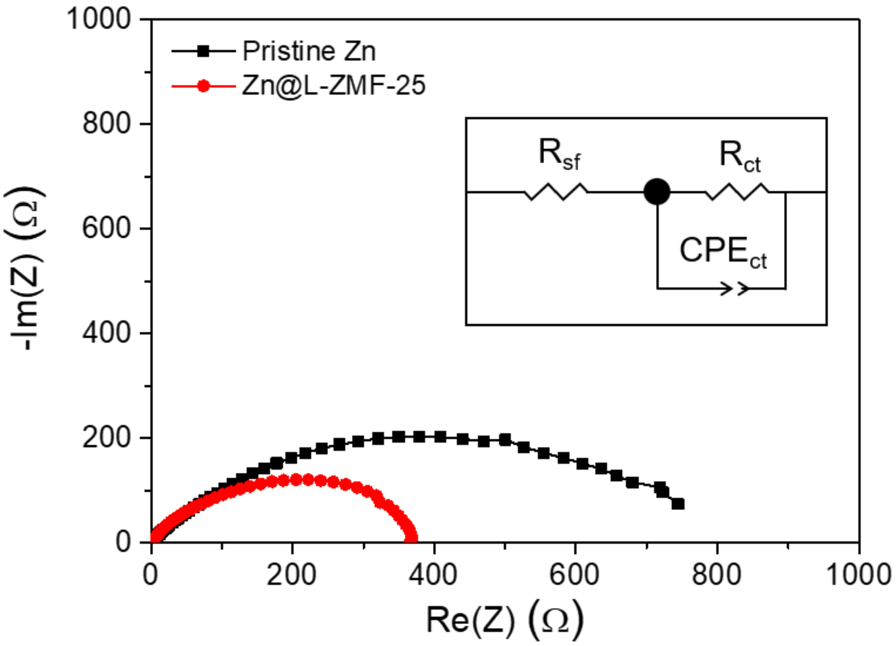

Fig. S9 Nyquist plots and equivalent circuit model for symmetric cells with the pristine Zn and Zn@L-ZMF-25 electrodes before the cycling test

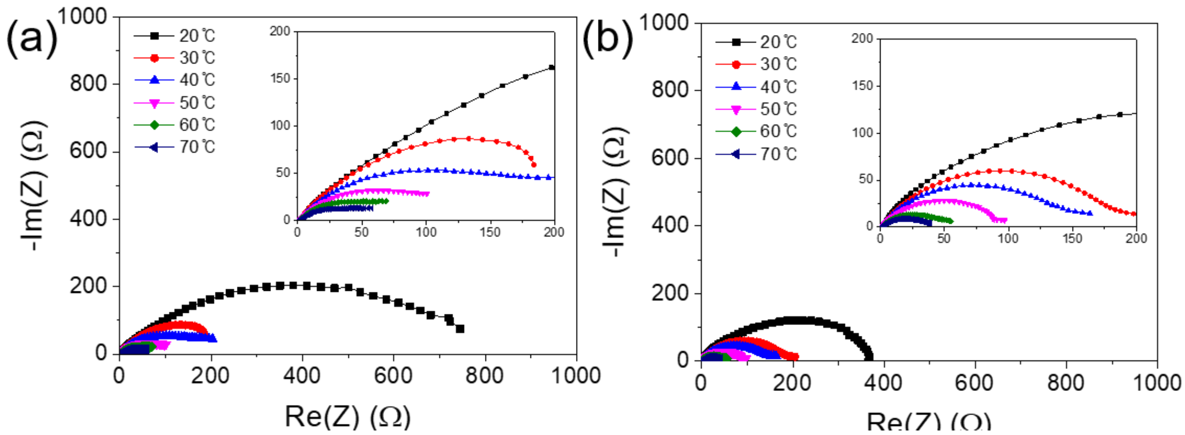

Fig. S10 Nyquist plots of symmetric cells with (**a**) pristine Zn and (**b**) Zn@L-ZMF-25 electrodes under various temperatures

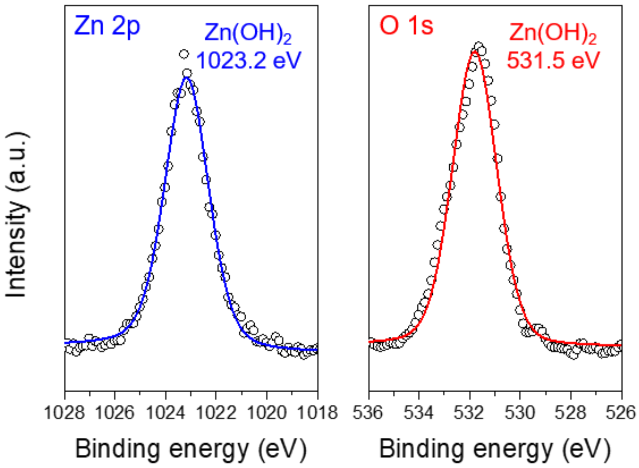

Fig. S11 Gaussian fitting of XPS spectra at the surface of pristine Zn metal electrodes after 250 galvanostatic cycles at a current density of 1.0 mA cm^{-2} with an areal capacity of 1.0 mAh cm^{-2}

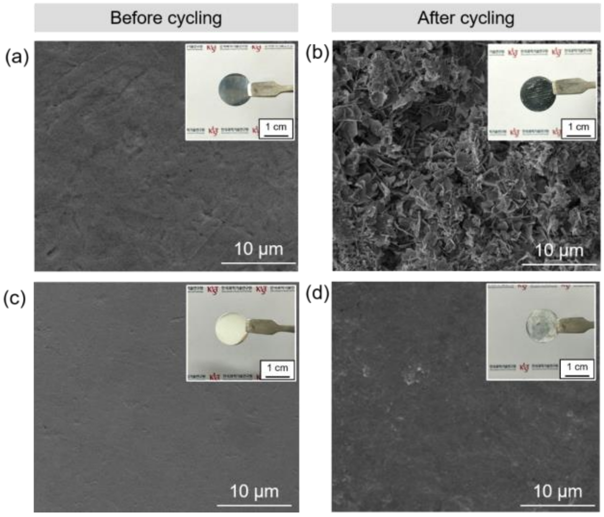

Fig. S12 Top-view SEM images of (**a, b**) pristine Zn and (**c, d**) Zn@L-ZMF-25 electrodes before and after 250 galvanostatic cycles at a current density of 1.0 mA cm^{-2} with an areal capacity of 1.0 mAh cm^{-2} (insets: photographs of the pristine Zn and Zn@L-ZMF-25 electrodes before and after galvanostatic cycling)

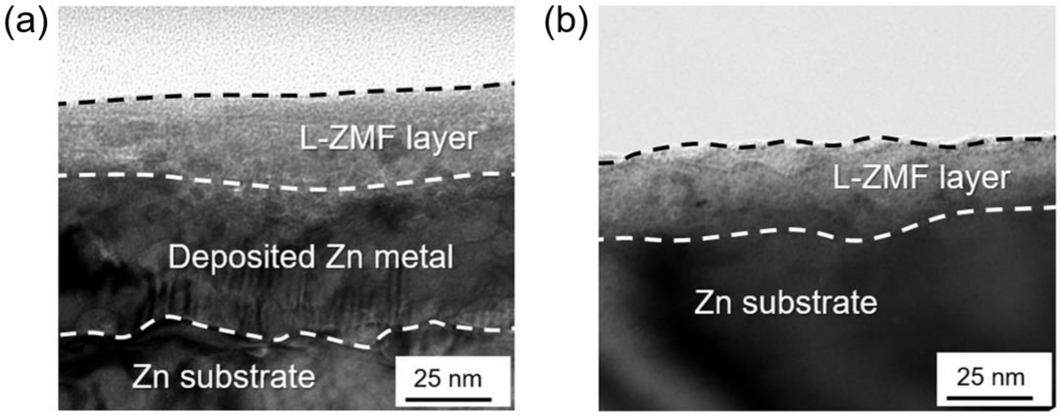

Fig. S13 Expanded cross-sectional TEM images of Zn@L-ZMF-25 electrodes (**a**) after Zn plating and (**b**) after stripping of plated Zn

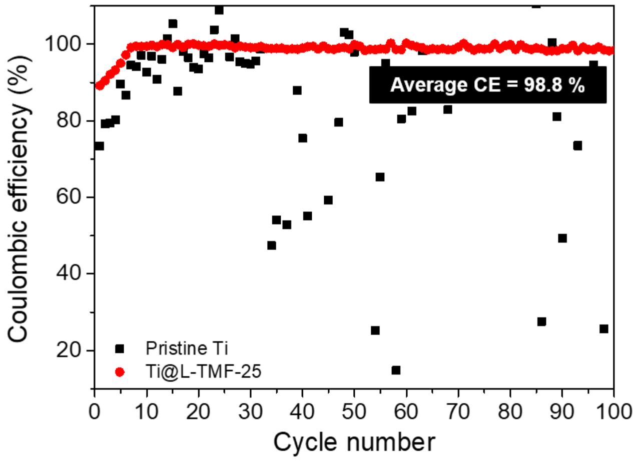

Fig. S14 Coulombic efficiency (CE) of Zn plating and stripping on pristine Ti and Ti@L-TMF-25 electrodes at a current density of 1.0 mA cm^{-2}

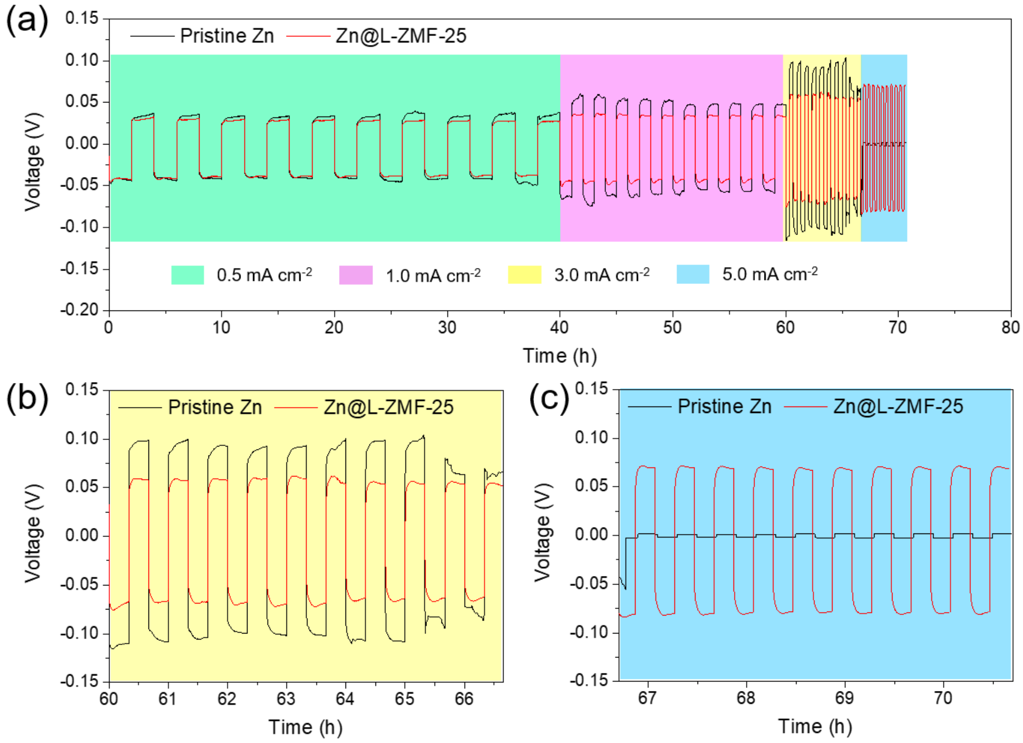

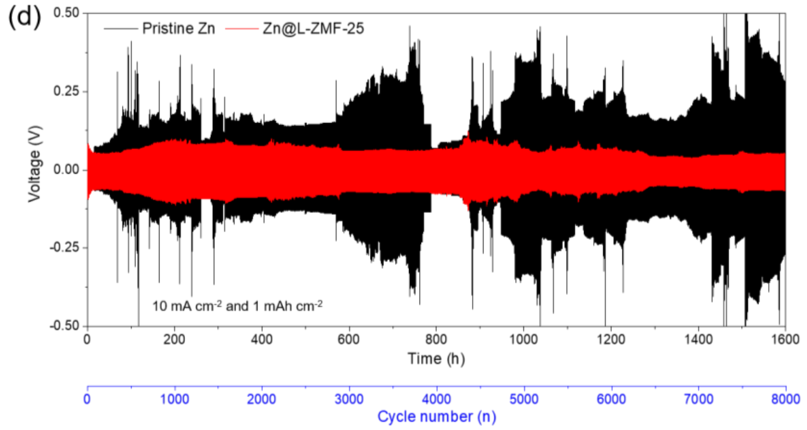

Fig. S15 (**a**) Voltage profiles of the galvanostatic cycling of symmetric cells with pristine Zn and Zn@L-ZMF-25 electrodes at various current densities with a constant areal capacity of 1.0 mAh cm^{-2}. Expanded voltage profile at a current density of (**b**) 3.0 and (**c**) 5.0 mA cm^{-2}. (**d**) Long-term Zn plating and stripping performance of symmetric cells with pristine Zn and Zn@L-ZMF-25 electrodes at a current density of 10.0 mA cm^{-2} with an areal capacity of 1.0 mAh cm^{-2}

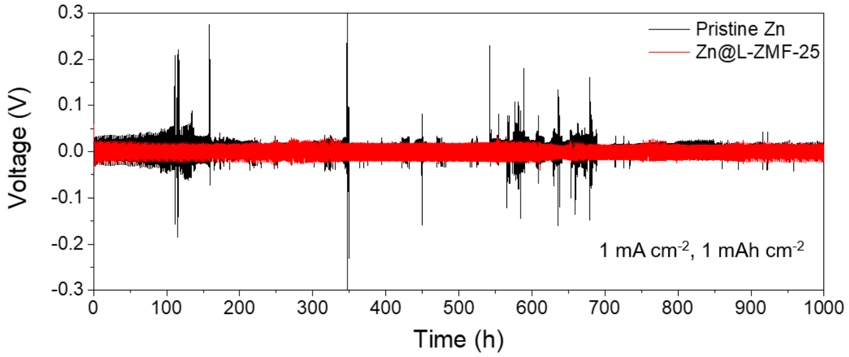

Fig. S16 Long-term Zn plating and stripping performance of symmetric cells with pristine Zn and Zn@L-ZMF-25 electrodes in 1 mol L^{-1} $ZnSO_4$ electrolyte at a current density of 1.0 mA cm^{-2} with an areal capacity of 1.0 mAh cm^{-2}

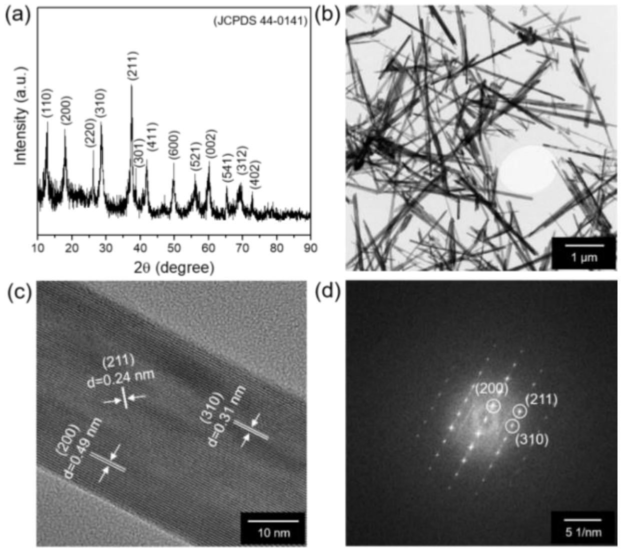

Fig. S17 (**a**) XRD pattern, (**b, c**) TEM images, and (**d**) fast Fourier transform (FFT) pattern of the synthesized α-MnO_2

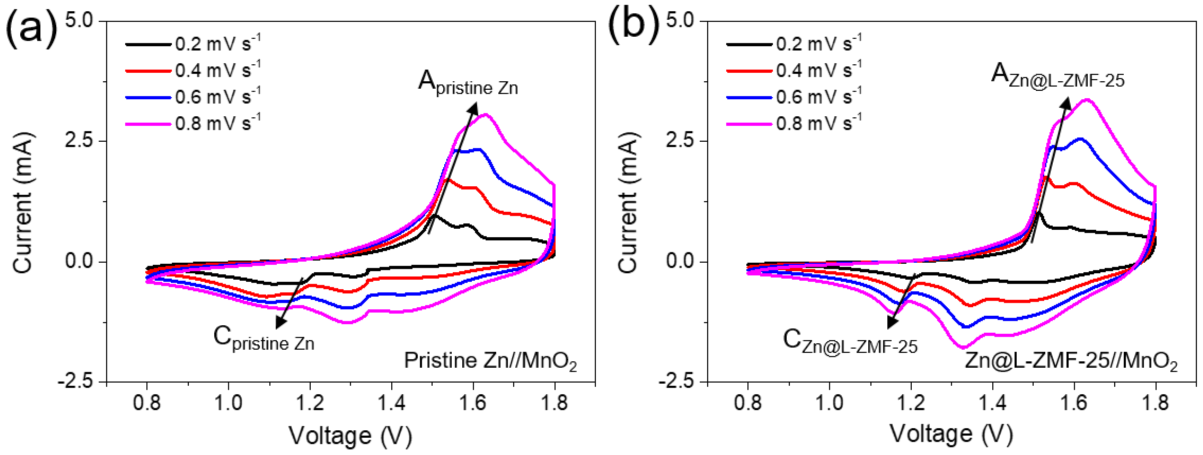

Fig. S18 Cyclic voltammetry curves of Zn/MnO_2 cells with (**a**) pristine Zn and (**b**) Zn@L-ZMF-25 anodes at various scan rates

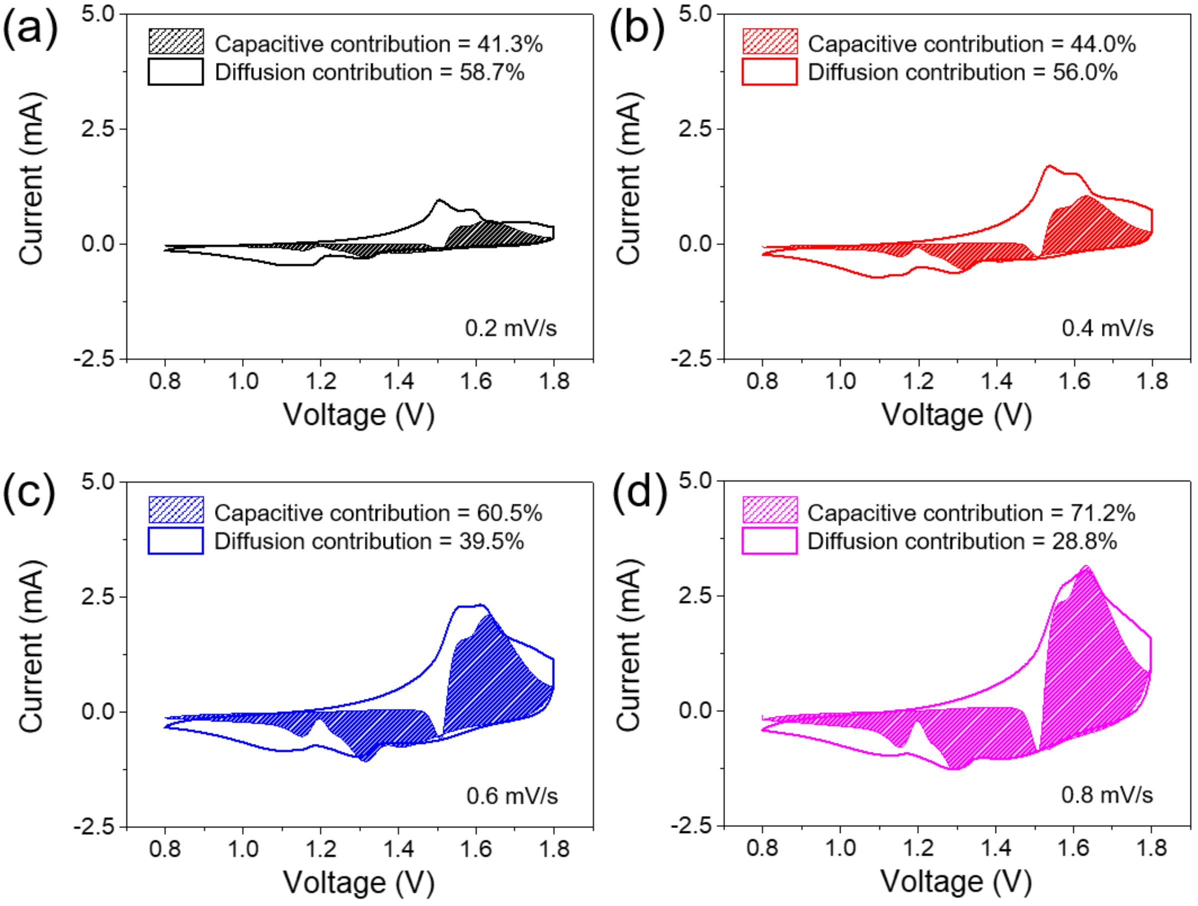

Fig. S19 Capacitive and diffusion-controlled behaviors of the pristine Zn/MnO_2 cell at different scan rates

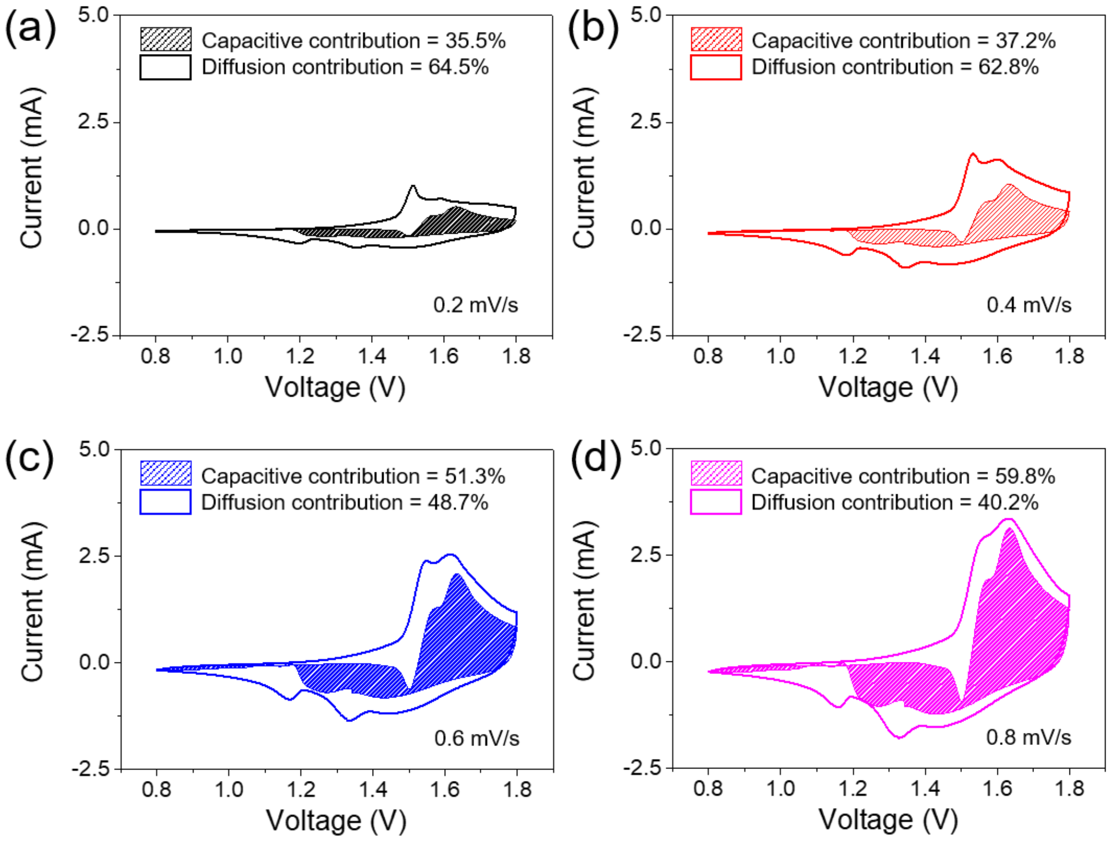

Fig. S20 Capacitive and diffusion-controlled behaviors of the Zn@L-ZMF-25/MnO_2 cell at different scan rates

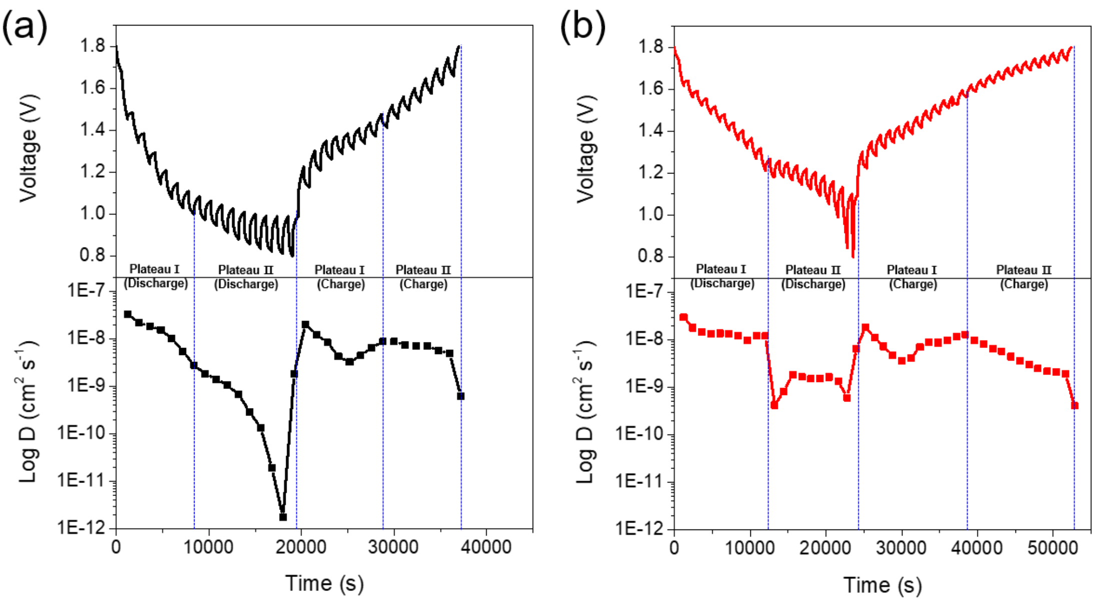

Fig. S21 Galvanostatic intermittent titration technique (GITT) and diffusion coefficient curves of Zn/MnO_2 cells with (**a**) pristine Zn and (**b**) Zn@L-ZMF-25 anodes at a constant current of 50 mA g^{-1} for an interval time of 10 min and suspended time of 10 min in open-circuit state

Note for Fig. S21: The diffusion coefficient was calculated using the GITT based on Eq. S2 [S1].

$$D = \frac{4L^2}{\pi t} \times \left(\frac{\Delta E_s}{\Delta E_t}\right)^2 \qquad \text{(S2)}$$

where L is the ion diffusion length (cm), which is generally identical to the thickness of the electrode, and t is the duration of the current pulse (s). $\triangle E_t$ is the voltage change for the applied constant current excluding the iR drop, and $\triangle E_s$ is the iR voltage drop due to the current pulse.

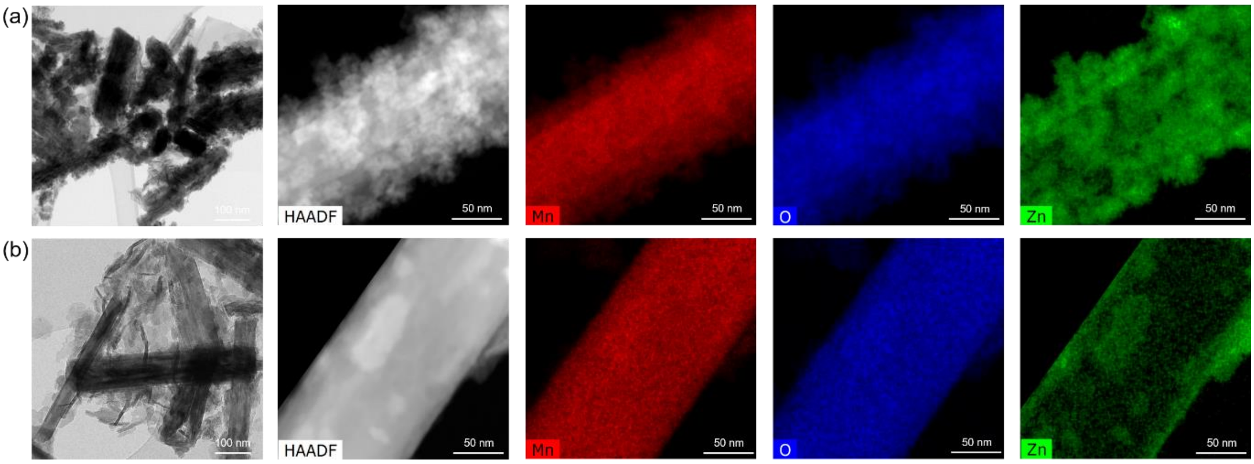

Fig. S22 Bright/dark field TEM images with the corresponding elemental maps of the α-MnO_2 cathodes of Zn/MnO_2 cells with (**a**) pristine Zn and (**b**) Zn@L-ZMF-25 anodes after 3000 galvanostatic cycles

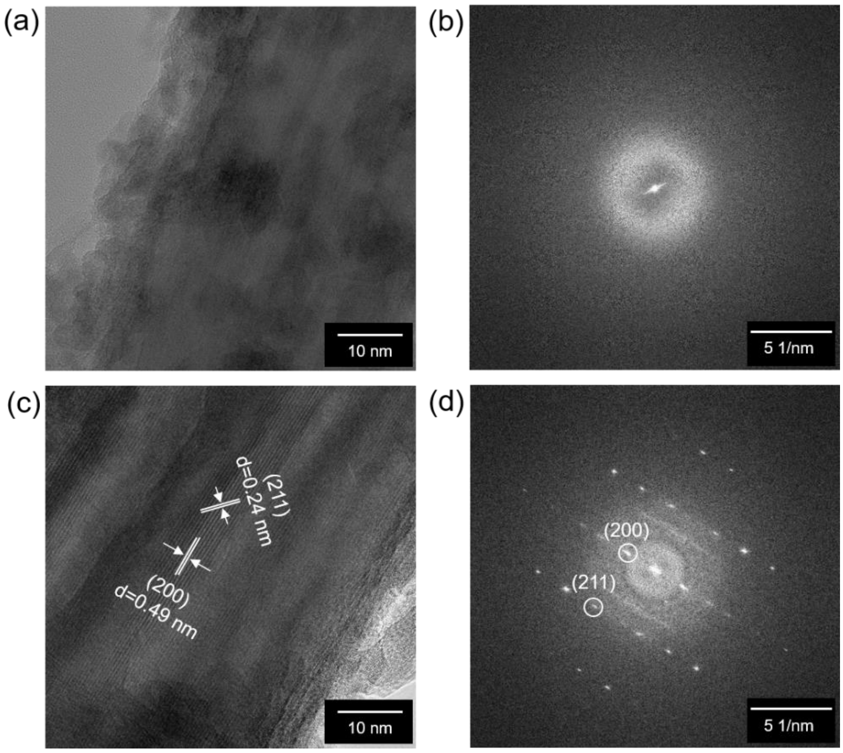

Fig. S23 High-resolution TEM images with the corresponding FFT patterns of the α-MnO_2 cathode of Zn/MnO_2 cells with (**a, b**) pristine Zn and (**c, d**) Zn@L-ZMF-25 anodes after 3000 galvanostatic cycles

Table S1 Corrosion potential (E_{corr}) and current (i_{corr}) of the pristine Zn, Zn@L-ZMF-13, Zn@L-ZMF-25, and Zn@L-ZMF-40 cells

	**Pristine Zn**	**Zn@L-ZMF-13**	**Zn@L-ZMF-25**	**Zn@L-ZMF-40**
E_{corr} (V)	-1.660	-1.655	-1.647	-1.647
i_{corr} (mA)	0.093	0.081	0.050	0.049

Table S2 Charge transfer resistance (R_{ct}) and transference number ($t_{Zn^{2+}}$) of the pristine Zn, Zn@L-ZMF-13, Zn@L-ZMF-25, and Zn@L-ZMF-40 electrodes

	**Pristine Zn**	**Zn@L-ZMF-13**	**Zn@L-ZMF-25**	**Zn@L-ZMF-40**
$\mathbf{R_{ct}}$ (Before polarization)	1903	1459	1678	1927
$\mathbf{R_{ct}}$ (After polarization of 50th sec)	1933	1470	1673	1911
$\mathbf{R_{ct}}$ (After polarization of 4000th sec)	2100	1706	1492	1635
$t_{Zn^{2+}}$ (50th sec)	0.406	0.537	0.488	0.410
$t_{Zn^{2+}}$ (4000th sec)	0.295	0.380	0.454	0.385

Table S3 Anodic resistances for interface (R_{sf}) and charge transfer (R_{ct}) of symmetric cells with the pristine Zn and Zn@L-ZMF-25 electrodes at various temperatures

**Temperature (°C)**	**Pristine Zn**		**Zn@L-ZMF-25**	
	$\mathbf{R_{sf}}$	$\mathbf{R_{ct}}$	$\mathbf{R_{sf}}$	$\mathbf{R_{ct}}$
20	20.6	756.9	12.2	376.3
30	5.48	353.6	2.36	190.0
40	1.40	195.3	0.59	151.0
50	0.29	114.7	0.22	93.1
60	0.23	77.8	0.15	52.3
70	0.15	40.2	0.12	36.4

Table S4 Comparison of the reversibility of Zn plating and stripping in the Zn-based symmetric cell with those in recent literature

**Coating material**	**Electrolyte**	**Current density (mA cm^{-2})**	**Areal capacity (mAh cm^{-2})**	**Overpotential (mV)**	**Cycling lifetime**	**Refs.**
Porous ZnO	2M $ZnSO_4$/ 0.1M $MnSO_4$	1.0<br>5.0	0.25<br>1.25	42<br>43	1000 cycles (5.0 mA cm^{-2}/1.25 mAh cm^{-2})	[S1]
Glass fiber@ Collagen hydrolysate	1M $ZnSO_4$	1.0	1.0	180	29 cycles (1.0 mA cm^{-2}/1.0 mAh cm^{-2})	[S2]

Polyamide/ $Zn(SO_3CF_3)_2$	2M $ZnSO_4$	0.5<br>10.0	0.25<br>10.0	100<br>100	8000 cycles (0.5 mA cm^{-2}/0.25 mAh cm^{-2})	[S3]
ZIF-8	2M $ZnSO_4$	0.25	0.05	60	425 cycles (0.25 mA cm^{-2}/0.05 mAh cm^{-2})	[S4]
Indium	2M $ZnSO_4$	4.0	1.0	40	800 cycles (4.0 mA cm^{-2}/1.0 mAh cm^{-2})	[S5]
H substituted graphdiyne	2M $ZnSO_4$	1.0<br>2.0	0.1	40<br>60	6000 cycles (0.5 mA cm^{-2}/0.1 mAh cm^{-2})	[S6]
Porous kaolin $Al_2Si_2O_5(OH)_4$	2M $ZnSO_4$/ 0.1M $MnSO_4$	4.4	1.1	70	1600 cycles (4.4 mA cm^{-2}/1.1 mAh cm^{-2})	[S7]
Mxene ($Ti_3C_2T_x$)	2M $ZnSO_4$	0.2<br>5.0	0.2<br>1.0	47<br>112	400 cycles (0.2 mA cm^{-2}/0.2 mAh cm^{-2})	[S8]
ZrO_2	2M $ZnSO_4$	1.0	1.0	70	3600 cycles (0.25 mA cm^{-2}/0.125 mAh cm^{-2})	[S9]
ZnS	1M $ZnSO_4$	2.0	2.0	49	250 cycles (2.0 mA cm^{-2}/2.0 mAh cm^{-2})	[S10]
**MgF_2 thin film with gradual Zn doping (Zn@L-ZMF-25)**	**1M $ZnSO_4$**	**1.0**	**1.0**	**17.2**	**500 cycles (1.0 mA cm^{-2}/1.0 mAh cm^{-2}) 8000 cycles (10.0 mA cm^{-2}/1.0 mAh cm^{-2})**	**This work**
	**1M $Zn(SO_3CF_3)_2$**	**0.5<br>1.0<br>3.0<br>5.0<br>10.0**		**24.9<br>27.2<br>55.5<br>69.3<br>99.7**		

Table S5 Comparison of capacity retention of the Zn/MnO_2 cell with those in recently published papers

Anode	Cathode	Electrolyte	Working voltage (V)	Current density (A g^{-1})	Initial specific capacity (mAh g^{-1})	Capacity retention (Cycle number)	Refs.
Zn@CNT	CNT-MnO_2	2M $ZnSO_4$/	1.0–1.8	2.3	187.0	88.7%	[S11]

Zn@PA-$Zn(CF_3SO_3)_2$	α-MnO_2	2M $ZnSO_4$/	0.8–1.8	2.0	141.0	88.0%	[S3]
Zn foil	Zn^{2+} installed	2M $ZnSO_4$/	1.0–1.9	3.0	115.8	72.8%	[S12]
Zn@ZnO	α-MnO_2	2M $ZnSO_4$/	0.8–1.8	1.0	100.0	88.2%	[S1]
Zn foil	α-MnO_2-TiN/TiO	1M $Zn(CH_3CO_2$	0.8–2.0	0.1	304.6	79.7%	[S13]
Zn@ZnS	Carbon cloth	1M $ZnSO_4$/	1.1–1.8	1.54	125.8	87.6%	[S10]
Zn@Kaolin $(Al_2Si_2O_5(OH)_4$	α-MnO_2	2M $ZnSO_4$/	0.8–1.8	0.5	217.1	87.5%	[S7]
**Zn@L-ZMF-25**	**α-MnO_2**	**1M $Zn(SO_3CF_3)_2$ /0.1M $MnSO_4$**	**1.0–1.8**	**2.0**	**98.8**	**97.5% (1000) 84.0% (3000)**	**This work**

Supplementary References

[S1] X. Xie, S. Liang, J. Gao, S. Guo, J. Guo et al., Manipulating the ion-transfer kinetics and interface stability for high-performance zinc metal anodes. Energy Envivon. Sci. **13**(2), 503-510 (2020). http://doi.org/10.1039/C9EE03545A

[S2] J. Zhi, S. Li, M. Han, P. Chen, Biomolecule-guided cation regulation for dendrite-free metal anodes. Sci. Adv. **6**(32), abb1342 (2020). http://doi.org/10.1126/sciadv.abb1342

[S3] Z. Zhao, J. Zhao, Z. Hu, J. Li, J. Li et al., Long-life and deeply rechargeable aqueous Zn anodes enabled by a multifunctional brightener-inspired interphase. Energy Envivon. Sci. **12**(6), 1938-1949 (2019). http://doi.org/10.1039/C9EE00596J

[S4] X. Pu, B. Jiang, X. Wang, W. Liu, L. Dong et al., High-performance aqueous zinc-ion batteries realized by MOF materials. Nano-Micro Lett. **12**, 152 (2020). http://doi.org/10.1007/s40820-020-00487-1

[S5] K. Hu, X. Guan, R. Lv, G. Li, Z. Hu et al., Stabilizing zinc metal anodes by artificial solid electrolyte interphase through a surface ion-exchanging strategy. Chem. Eng. J. **396**, 125363 (2020). http://doi.org/10.1016/j.cej.2020.125363

[S6] Q. Yang, Y. Guo, B. Yan, C. Wang, Z. Liu et al., Hydrogen-substituted graphdiyne ion tunnels directing concentration redistribution for commercial-grade dendrite-Free zinc anodes. Adv. Mater. **32**(25), 2001755 (2020). http://doi.org/10.1002/adma.202001755

[S7] C. Deng, X. Xie, J. Han, Y. Tang, J. Gao et al., A sieve-functional and uniform-porous kaolin layer toward stable zinc metal anode. Adv. Funct. Mater. **30**(21), 2000599 (2020). http://doi.org/10.1002/adfm.202000599

[S8] N. Zhang, S. Huang, Z. Yuan, J. Zhu, Z. Zhao et al., Direct self-assembly of MXene on Zn anodes for dendrite-free aqueous zinc-ion batteries. Angew. Chem. Int. Ed. **60**(6), 2861-2865 (2021). http://doi.org/10.1002/anie.202012322

[S9] P. Liang, J. Yi, X. Liu, K. Wu, Z. Wang et al., Highly reversible Zn anode enabled by controllable formation of nucleation sites for Zn-based batteries. Adv. Funct. Mater. **30**(13), 1908528 (2020). http://doi.org/10.1002/adfm.201908528

[S10] J. Hao, B. Li, X. Li, X. Zeng, S. Zhang et al., An in-depth study of Zn metal surface chemistry for advanced aqueous Zn-ion batteries. Adv. Mater. **32**(34), 2003021 (2020). http://doi.org/10.1002/adma.202003021

[S11] Y. Zeng, X. Zhang, R. Qin, X. Liu, P. Fang et al., Dendrite-free zinc deposition induced by multifunctional CNT frameworks for stable flexible Zn-ion batteries. Adv. Mater. **31**(36), 1903675 (2019). http://doi.org/10.1002/adma.201903675

[S12] J. Wang, J. Wang, H. Liu, C. Wei, F. Kang, Zinc ion stabilized MnO_2 nanospheres for high capacity and long lifespan aqueous zinc-ion batteries. J. Mater. Chem. A **7**(22), 13727-13735 (2019). http://doi.org/10.1039/C9TA03541A

[S13] S. Chen, R. Lan, J. Humphreys, S. Tao, Salt-concentrated acetate electrolytes for a high voltage aqueous Zn-MnO_2 battery. Energy Storage Mater. **28**, 205-215 (2020). http://doi.org/10.1016/j.ensm.2020.03.011
